# Exploring the Vertical Transmission of Exosomes in Diagnostic and Therapeutic Targets for Pregnancy Complications

**DOI:** 10.1021/acsbiomaterials.5c00119

**Published:** 2025-06-13

**Authors:** Shrikrishna Bhagat, Rakshith Hanumanthappa, Ketki Bhokare, Neelabh Datta, Nidhi Vastrad, M. David, N. Maharaj, Krishnan Anand

**Affiliations:** † 29722 Institute of Life Sciences, Bhubaneswar 751023, Odisha, India; ‡ JSS Banashankari Arts, Commerce, and SK Gubbi Science College, Karnatak University, Dharwad 580004, Karnataka, India; § Department of Oral and Maxillofacial Pathology, Vidarbha Youth Welfare Society’s Dental College and Hospital, Amravati 444602, India; ∥ School of Biology, Indian Institute of Science Education and Research, Thiruvananthapuram (IISER-TVM), Thiruvananthapuram 695551, Kerala, India; ⊥ Department of Obstetrics and Gynaecology, School of Clinical Medicine, Faculty of Health Sciences, 37702 University of the Free State, Bloemfontein 9300, South Africa; # Precision Medicine and Integrated Nano-Diagnostics (P-MIND) Research Group, Office of the Dean, Faculty of Health Sciences, University of the Free State, Bloemfontein 9300, South Africa

**Keywords:** placental EVs, exosomes, preeclampsia, gestational diabetes, immunomodulation, biomarker discovery

## Abstract

During pregnancy, the mother-placenta relationship involves intricate and dynamic exchanges. Over the course of gestation, the maternal body encounters numerous fetal materials secreted by the placenta, such as hormones, growth factors, and extracellular vesicles like exosomes. These exosomes are carriers of key biomolecules, including proteins, lipids, nucleic acids (DNA), and microRNA (miRNA), capable of influencing maternal cellular activity. Although the exact functions of placental exosomes during pregnancy remain under investigation, existing research indicates that they contribute significantly to normal placental growth and maternal immune tolerance, both of which are vital for sustaining a healthy pregnancy. The involvement of exosomes in the etiology and progression of pregnancy complications is also under investigation. Variations in the quality and quantity of placenta-derived exosomes, their concentration in maternal plasma, and their composition and bioactivity have been linked to complications such as gestational diabetes, preeclampsia, and maternal infections. There is considerable interest not only in understanding the role of placenta-derived exosomes in both normal and complicated pregnancies but also in their potential as biomarkers and therapeutic targets. Progress in this field depends on using specific and well-characterized methodologies and techniques to precisely determine the role of exosomes in pregnancy complications and their clinical utility. This review emphasizes the significance of placenta-derived exosomes in pregnancy, focusing on their interaction with the maternal system. Additionally, it explores new techniques and ideas for analyzing placental exosomes as potential biomarkers for the early diagnosis of pregnancy complications.

## Graphical abstract

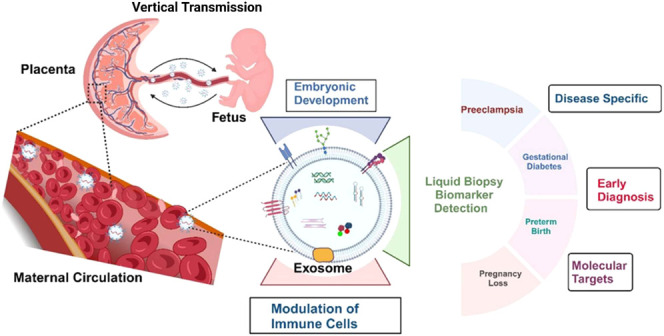

## 1 Introduction

Pregnancy is a complex biological process, marked by numerous molecular and cellular interactions that occur during each trimester to support, maintain, and facilitate the successful delivery of the baby.^1^ It is a state that alters normal physiological conditions, usually without negative repercussions to the mother. Some women experience health problems during pregnancy, even if they were healthy before, and these intricacies can harm both the mother and the fetus, turning the pregnancy into a high-risk situation. According to the United Nations International Children’s Emergency Fund (UNICEF), one pregnant woman or a newborn dies every 11 seconds worldwide. This significant risk to the health of pregnant women and babies increases dramatically during pandemics.^2^ Maternal health complications represent a significant public health challenge, endangering fetal development and placing substantial socioeconomic strain due to the increased demand for healthcare and social support services.^3^ Recently, COVID-19 has been proven to be correlated with potential adverse outcomes for both the mother and the baby.^4^ Many lifestyle behaviors contribute to pregnancy difficulties, as most substances of abuse, alcohol, etc., easily pass the placenta and can impact fetal brain development^5^ as well as environmental factors like heat stress^6,7^ and pollution,^8^ which trigger adverse pregnancy consequences. Neonatal mortality is high due to abnormal fetal development and growth illnesses such as neural tube abnormalities, congenital heart disease, and numerous deformities.^9^ Pregnancy complications include conditions that occur during pregnancy, such as gestational diabetes,^10^ gestational hypertension, preeclampsia,^11^ preterm birth, and fetal growth limitation. However, the burden is immense, with approximately 3–5% of women worldwide experiencing preeclampsia, a new-onset multisystemic hypertensive disorder of pregnancy.^11^

Despite significant improvements in monitoring and prevention in other areas of healthcare, the etiology of complications associated with pregnancy still remains unknown. Early detection of pregnancy-related complications and abnormal fetal development is largely dependent on standard hematological assessments and ultrasound imaging techniques. One key challenge with many current therapies is their inability to precisely target the maternal or fetal compartments or the placental interface, depending on the condition, which limits their effectiveness and may lead to unwanted side effects.^12^ During gestation, the placenta secretes various molecules that modify maternal physiology to meet the fetus’s needs. It can also impact the mother’s physiological processes through extracellular vesicles (EVs).^13^ Reports suggest that exosomes play a role in paracrine communication between fetal and maternal tissues. In pregnancies with complications, this form of cellular communication contributes to the presentation of disease symptoms, as the release of exosomes is influenced by the surrounding maternal microenvironment.^14^ The precise origin, cargo, and functions of exosomes in maternal circulation during pregnancy are still under investigation, and further research is needed to fully understand their role in both normal and complicated pregnancies. The present review explores in detail the role of exosomes during gestation and their trafficking into the maternal circulation, with emphasis on immune and metabolic adaptations to regulate different pregnancy complications and their implications in clinical use.

## 2 Exosomes and Their Biogenesis

Exosomes, often described as molecular messengers, are nanosized vesicles that belong to a broader category of extracellular vesicles (EVs). Derived from nearly every cellular compartment, exosomes influence the function and fate of the recipient cells. These phospholipid vesicles follow a specific pathway that allows them to mediate communication between cells, thereby regulating a variety of physiological processes. The endocytosis of molecular cargo initiates this pathway and internalizes diverse biological substances including proteins, lipids, nucleic acids, and signaling molecules. Exosome formation within the endosomal system occurs as early endosomes transition into late endosomes, characterized by the loss of RAB5A, RAB4, RAB11, and RAB22 proteins and the acquisition of late endosomal markers like RAB7 and RAB9A.^15,16^ The RAB family of G-proteins controls different functions, wherein the RAB GTPases play a crucial role in regulating every facet of intracellular vesicle trafficking. Exosomes form intraluminal vesicles (ILVs) in late endosomes via inward budding of endosomal multivesicular bodies (MVBs), which either degrade or fuse with the plasma membrane, releasing ILVs as exosomes.^17,18^ The biogenesis of exosomes is controlled by activating cell-specific receptors and signaling pathways. Reports have indicated that syndecan heparan sulfate proteoglycans and their cytoplasmic adaptor syntenin control the formation of exosomes and play a key role in membrane transport and cell signaling.^19^ The Endosomal-Sorting Complex required for Transport (ESCRT), an intricate protein molecule consisting of ubiquitous subunits, ESCRT- 0, ESCRT- I, ESCRT- II, and ESCRT- III is responsible for sorting molecules into ILVs and facilitating exosomal cargo sorting and vesicle budding.^20^ ESCRT-0 recognizes ubiquitin-tagged proteins, forming a complex with ESCRT-I and ESCRT-II, which associates with ESCRT-III to bud vesicles. Vps4 provides energy for ESCRT-III to cleave buds and produce ILVs, indicating the existence of an ESCRT-independent ILV formation pathway.^21,22^ The ESCRT-independent mode is an ancillary pathway involving lipid raft-associated tetraspanins like CD9, CD63, and CD81 and heat shock proteins, which facilitate membrane budding and cargo sorting, also leverage ceramide and sphingolipid metabolism^23^ promoting membrane curvature and vesicle release for cellular communication and molecular transport.^24^ Exosomes carry many macromolecules such as DNA, lipids, transcriptional regulators, signaling proteins, and diverse RNA species. Regulatory RNAs, such as miRNAs and lncRNAs, bind to target genes and mediate signaling pathways that influence immune system dynamics. Additionally, they contain tRNAs, snRNAs, snoRNAs, piwi-interacting RNAs (piRNAs), and mtDNA, dsDNA, and ssDNA.^20^ Furthermore, exosomes contain enzymes like GTPases, calcium-dependent phospholipid-binding proteins called Annexins, and membrane-associated proteins known as Flotillin. These include proteins that occupy endosomal multivesicle formation, i.e., Alix and TSG101. Heat shock proteins such as Hsc70 & Hsp 90 along with the tetraspanins CD9, CD63, CD81, and CD82, regulate exosome cargo sorting and release.^25^ The therapeutic cancer target CD151, or PETA-3, has shown that it is associated with sperm cells and mediates exosome biogenesis, playing vital roles in cellular processes via integrin and nonintegrin proteins. Approximately 4400 exosomal proteins bind directly to target cells.^26,27^ Phospholipases, raft-associated lipids such as cholesterol, ceramide, sphingolipids, and phosphoglycerides with long and saturated fatty-acyl chains are also present.^28^ The overall biogenesis process of the exosomes and MVs from the cells is depicted in Figure 1.

**Figure 1:**
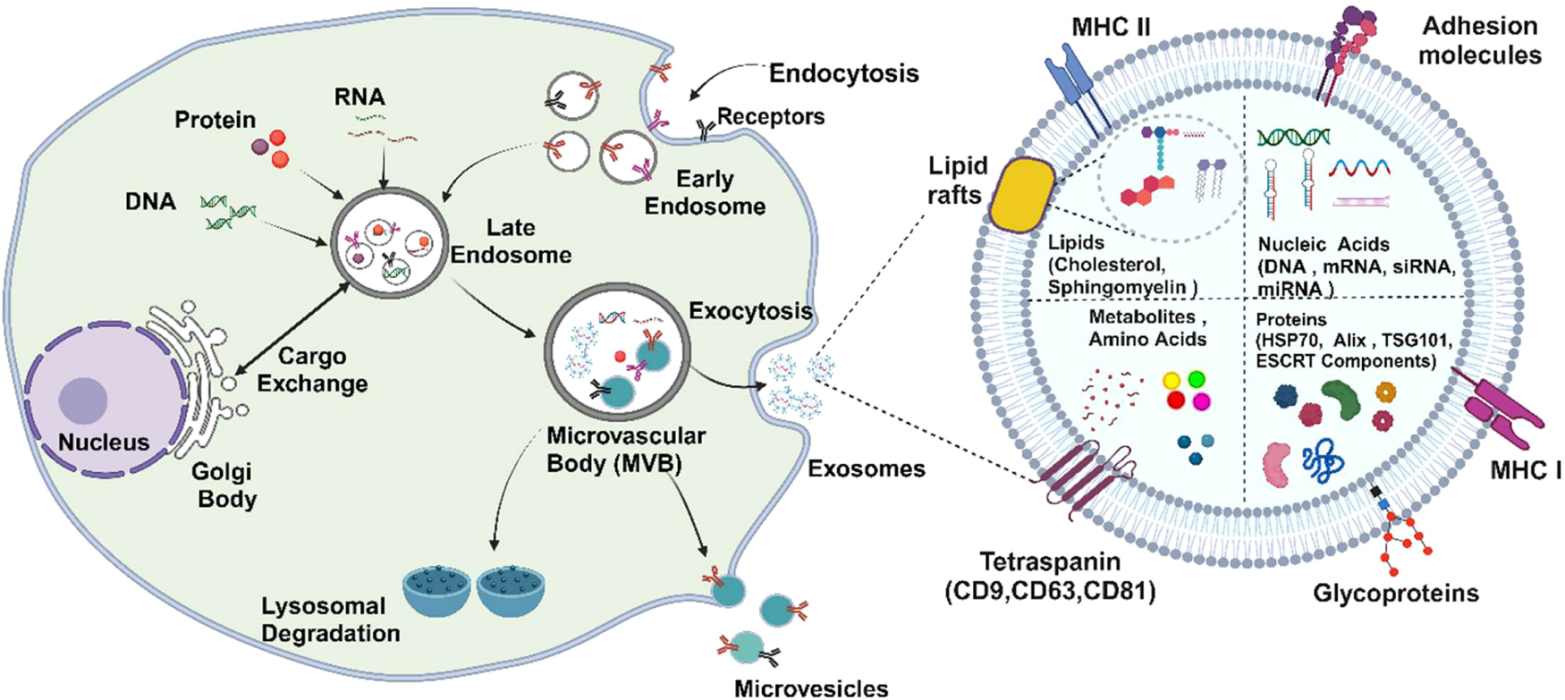
Schematic overview of biogenesis of the exosomes from the cell and the detailed structure of the exosome (created with Biorender.com).

### 2.1 Exosomes as Mediators of Cell-to-Cell Communication in Pregnancy

Cells utilize various mechanisms to communicate, including the release of soluble factors, direct adhesion interactions, tunneling nanotubules, etc., for unmediated association, thus facilitating cell-to-cell coordination. Exosomes have emerged as significant contributors to this dynamic process, uncovering a previously underestimated dimension of cellular communication.^29^ Initially believed as a cellular apparatus for disposing of debris, exosomes are now known to transport crucial molecules and are involved in the transfer of genetic information and biological signals to recipient cells via surface molecules such as exosomal integrins, engaging through membrane fusion, endocytosis or receptor binding.^30^ These tiny vesicles are present in maternal blood as well as in various biological fluids, crossing physiological barriers like the placental, endothelial, blood–brain barrier (BBB), and epithelial barrier.^31^ The placenta is a crucial organ in pregnancy that connects the fetus to the mother and releases exosomes into the maternal circulation, which are thought to originate from extravillous trophoblasts or syncytiotrophoblasts during the first trimester.^32^ Placenta-derived exosomes regulate cell migration and invasion, aiding placentation and maternofetal vascular development. The exosomes released are detectable at the onset of 6 weeks of pregnancy. This provides an opportunity to diagnose prenatal pregnancy complications and allows the development and assessment of suitable intervention strategies aimed at limiting acute unfavorable repercussions.^33^ The placental exosomes provide an essential conduit for communication between the placenta and maternal histological milieu in typical and advanced pregnancies.^34,35^ There is strong evidence indicating that exosomes are involved in intercellular communication by transporting bioactive molecules, which are essential for mediating interactions between maternal and fetal systems.^36^ Exosomes’ versatility as cell-to-cell communication mediators is further demonstrated by their possible involvement in pregnancy-related problems.^37^ For example, disorders such as Intrauterine Growth Restriction (IUGR) and Gestational Diabetes (GD) have been associated with aberrant exosomal cargo or release patterns.^38^

## 3 Mother-to-Child Transmission (Mtct) or Vertical Transmission

The human placenta releases various molecules and EVs to help the mother adapt to the developing fetus needs through vertical transmission.^13^ The growing fetus releases these vesicles, which comprise a variety of proteins and nucleic acids, including DNA, mRNAs, microRNAs, and long noncoding RNAs. The fetal and maternal cells internalize exosomes by a variety of routes like endocytosis, macropinocytosis, and phagocytosis mechanisms, and by fusing with endosomes, they release their cargos in the target cell’s cytoplasm. Also, the direct fusion of exosomal lipid and membrane protein with the recipient cell’s plasma membrane leads to internalization of exosomes. Furthermore, the selective uptake of the exosomes is carried out by the specific receptors present on the target cells.^39^ Studies have reported that microvesicles and exosomes, produced by the placenta in the first trimester, are absorbed by endothelial cells via phagocytosis and clathrin-mediated endocytosis, which is found to be organ-specific.^40^ To confirm this, researchers employed fluorescently tagged exosomes derived from fetal cells and subsequently injected into the amniotic fluid of pregnant mice, which modulate the maternal physiology to cause or adapt it to pregnancy-induced changes.^41^ Three different processes result in the release of exosomes: (i) endocytic vesicles’ plasma membrane penetration, (ii) endosomal membranes budding inward to form MVBs and (iii) MVBs fusing with the plasma membrane to release exosomal contents.^36^ Lipid compounds like ceramides and phosphatidic acid regulate exosome production and release, with the size influenced by their origin and lipid bilayer. Exosomes derived from the placenta can be detected in maternal blood as early as 6 weeks of gestation, and their concentration increases as the pregnancy progresses. microRNA-30d-5p, found in placental exosomes, has been shown to induce macrophage polarization into alternatively activated (M2) macrophages, promoting trophoblast migration and invasion while inhibiting endothelial cell tube formation and migration.

The placenta secretes exosomes into the mother’s blood early in pregnancy, aiding communication with other maternal organs, though their exact functions remain unclear.^42^ In the course of implantation and placentation, fetal-maternal communication is facilitated by exosomes and other EVs. They adjust the mother’s uterine vasculature, encourage fetal vasculogenesis, preserve cellular metabolic homeostasis, control maternal responses, and get the uterus ready for birth control.^43^ Exosomes derived from the placenta can be detected in maternal blood as early as 6 weeks of gestation, and their concentration increases as the pregnancy progresses. Researchers have observed the discharge of placenta-derived exosomes in both healthy and abnormal pregnancies into the mother’s blood.^44^ The presence of distinct miRNAs or proteins allows for the identification of these placenta-specific exosomes.^45^ These vesicles enable non-invasive liquid biopsies by transmitting between fetal and maternal compartments. Placental alkaline phosphatase (PLAP), a specific membrane protein lacking 24 amino acids from the N-terminal region, is a unique surface marker and target for the purification of placental EVs.^34^ Recent methods using quantum dots and antibodies targeting PLAP and CD63 measure placental exosomes in maternal plasma, helping assess placental and fetal development.^46^ Fetal-derived exosomes cross over to the maternal side during pregnancy and may carry signals to the uterus and cervix. These minuscule vesicles have been linked to inflammatory processes in the past and may possibly have a role in the commencement of labor.^47^ Exosomes are paracrine mediators in mice that may induce birth without the need for systemic progesterone withdrawal, which is typically required to induce labor.^41^ Surprisingly, these exosomes are also essential for the growth and survival of the fetus within the mother. Exosomes derived from the placenta can be detected in maternal blood as early as 6 weeks of gestation, and their concentration increases as the pregnancy progresses. microRNA-30d-5p, found in placental exosomes, has been shown to induce macrophage polarization into alternatively activated (M2) macrophages, promoting trophoblast migration and invasion while inhibiting endothelial cell tube formation and migration.^48^

The number of placenta-derived exosomes in maternal plasma increases gradually during pregnancy, peaking at term.^49^ It is interesting to note that this growth starts much earlier than the whole development of intervillous circulation in the first trimester. Extravillous trophoblast invasion and proliferation are facilitated by factors such as hypoxia and hyperglycemia in the uterus, which cause syncytiotrophoblasts to produce more exosomes.^50^ For instance, placental exosomes have been shown to induce maternal immune tolerance by interacting with maternal immune cells, leading to the reprogramming of circulating monocytes.^51^ Exosomes have been studied via trophoblast cultures, chorionic villi explants, placental perfusion, and maternal plasma analysis. The research includes their roles in fetal–maternal exchange, implantation, and angiogenesis, with genetically modified mice used to differentiate exosome types.^52^ Remarkably, exosomes were transferred across placental boundaries from mother to fetus and from fetal to mother (Figure 2). Fetal exosomes have been found in mother plasma, which raises the possibility that they might be used as noninvasive pregnancy indicators.

**Figure 2:**
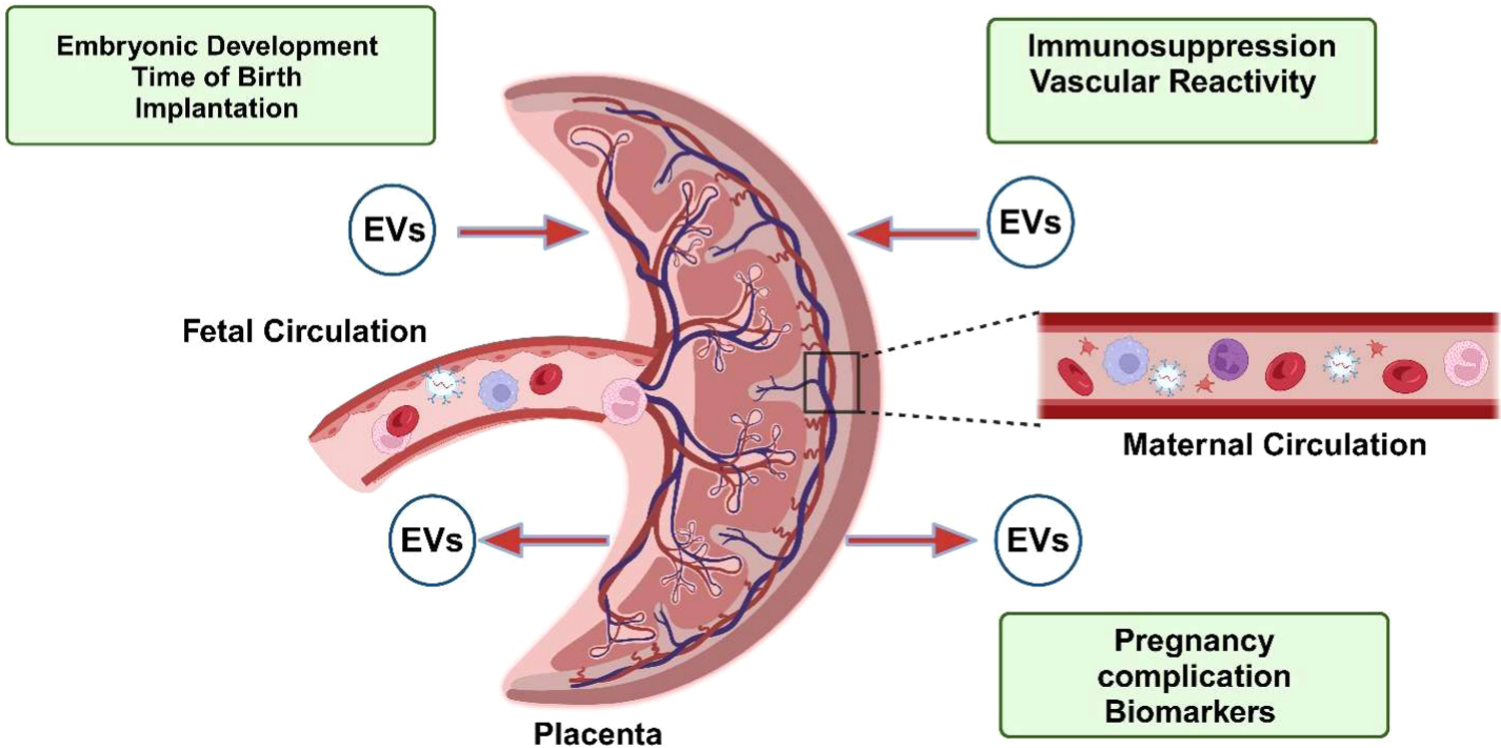
Schematic representation of maternal-to-fetal barrier of circulations and places of appearance of exosomes and development of the placenta concerning the maternal–fetal interface. The exosomes vertical transmission from tthe placenta to the maternal circulation and from the maternal side to the fetus (created with Biorender.com).

### 3.1 Roles of RNA and Exosomes during Vertical Transmission

The transfer of particular cargo molecules is intimately associated with exosomal bioactivity. Interestingly, noncoding RNAs (ncRNAs) make up a large fraction of transcripts in the human genome and are involved in complex regulatory networks.^53^ Researchers frequently utilize length to group ncRNAs. Long ncRNAs (lncRNAs) are longer than 200 ribonucleotides, whereas small ncRNAs, or microRNAs (miRNAs), are usually less than 200 ribonucleotides.^54^ Furthermore, a new type of noncoding RNAs called circular RNAs (circRNAs), has been identified. The miRNAs are integral to fundamental physiological processes such as cell migration, differentiation, and proliferation and are critically involved in the development and progression of various diseases, including cancer and immune-related disorders.^55^ These mature single-stranded miRNAs, processed through protein complexes, primarily function by utilizing the RNA-induced silencing complex (RISC) to induce post-transcriptional gene silencing, wherein RISC binds to recognition sequences in the 3′-untranslated region (UTR) of target mRNAs, leading to translational inhibition or mRNA instability; intriguingly, recent studies suggest that miRNAs might also enhance the expression of certain genes.^53,56^ Moreover, cells release miRNAs in a variety of ways, including as free molecules linked to protein complexes, encapsulated within exosomes and other EVs, and connected with lipoproteins.^57,58^ Because exosomal miRNAs are stable in bodily fluids and play essential roles in physiological and pathological processes, they have great potential for use in diagnostic and therapeutic applications. LncRNAs, which have a high degree of variability, are derived from genomic regions that code for proteins that are exonic, intergenic, or distal.^53,57^ Among their unique characteristics are 5′-splicing, 3′-polyadenylation, and thermodynamic stability. Changes in the expression levels of several lncRNAs have been linked to a variety of illnesses, even if the precise processes behind them are still unknown.^54^ Subcellular localization patterns are displayed by lncRNAs: cytoplasmic lncRNAs mainly affect post-transcriptional gene expression, whereas nuclear lncRNAs are related to epigenetic gene control. These molecules function as miRNA sponges, recruiters, competitors, and precursors of miRNA in their interactions with other biomolecules.^54,57^ Exosomal lncRNAs engage in intercellular communication by conveying data and causing alterations in nearby or remote cells^59^ and can be potential biomarkers for a variety of illnesses due to their tissue selectivity, greater concentration than other EVs, and resistance to enzyme destruction.^57^ Researchers are also exploring exosomes with miRNAs like miRNA-141 for delivering therapeutic nucleic acids, as these increase in maternal plasma during pregnancy.^60^ Placenta-associated miRNAs are found in the chromosome 19 miRNA cluster (C19MC), which is essential for placental-maternal transmission.^61^

Furthermore, exosomes help fetal defense against viral infections by transferring certain miRNAs to cells apart from placental ones. Table 1 describes the role of different exosomal RNAs in pregnancy. EVs generated from synctiotrophoblast have also been found to contain tRNA fragments.^62^ The adhesive potential of trophoblasts is increased by endometrial epithelial cells’ exosomes. Exosomal miR-30d upregulates integrin-related genes to enhance preimplantation embryo adhesion in a mouse model.^63^ Moreover, exosomal miR-520c-3p affects chorionic villous trophoblast cell invasion.^64^ Unique protein profiles throughout the cyclic and pregnant phases were found by evaluating the vesicle proteins in the uterine luminal fluid of sheep.^38^ These proteins may affect the results of implantation and fertility including prostaglandin synthase, lipoprotein lipase, gastrin-releasing peptide, and cathepsin L1. The meconium and extra-embryonic components that make up the mother–fetus interface are crucial in regulating the mother’s immune system to suit the growing fetus.^65^ Trophoblast cells activate the JNK and p38 signaling cascades in meconium macrophages by releasing ZEB2-AS1, an exosomal long noncoding RNA. This mechanism encourages an environment that is favorable for maternal–fetal immunological tolerance by encouraging macrophages to adopt a less inflammatory M2 phenotype. Once activated, these M2 macrophages respond by encouraging the growth and differentiation of trophoblast cells, which are vital to the advancement of a normal pregnancy. In contrast, mothers who have had repeated spontaneous miscarriages were discovered to have fewer M2 macrophages during metaphase; this may be because the trophoblast-derived exosomes contain less ZEB2-AS1.^66^ By limiting negative maternal immune responses and giving the placenta and fetal allografts immunological privileges in the uterus, apoptotic mechanisms which are aided by the secretion of FasL and TRAIL from early and term human placentas may help preserve pregnancy.^67^ These results emphasize the function of placental exosomes as complex channels for cellular communication, emphasizing their importance in establishing fetal immune privilege and preserving balance at the maternal–fetal nexus throughout gestation.^68^ The interaction of the blastocyst with a receptive uterus culminates in close contact with the endometrium during the crucial phase of gestation known as embryo implantation.^69^ This moment, which signifies the embryo’s successful implantation into the mother organism, is crucial to mammalian reproduction. According to research conducted in vitro, epithelial and stromal cells in the meconium may be able to change the local immunological environment through the consumption of placenta-derived exosomes (PEXOs), which would help to initiate and maintain pregnancy. At the maternal–fetal interface, exosomes play a critical role in the exchange of signals.^70^ Specifically, embryonic exosomes improve the embryo’s capacity to adapt, promote successful implantation, and initiate gestation. These vesicles also affect the expression of genes, including Bcl2, Bax, Casp3, and Tp53, that are linked to apoptosis in endometrial epithelial cells before implantation and bolster the expression of adhesion proteins postimplantation to support further attachment.^71^

**Table 1:** Different Physiological Roles of Exosomal RNA during Pregnancy

sr. no.	exosomal RNA	type	genes affected	biological functions	reference
1.	miR-451a	miRNA	ATF2	Cell cycle regulation, Proapoptotic protein regulation	^154^
2.	miR-185-5p	miRNA	RHOA, ATF6, and CDC42, MDM2, PKD1	Cell Migration, Cytoskeletal Formation, Cell Motility, ER Stress	^204,205^
3.	miR-4535-3p	miRNA	CD44, LHFPL3, KDM1B, RNF19A, C19orf82, and FKBP4	Regulation, Tumor Suppression, Diagnostic Biomarker	^206^
4.	miR-1-3p	miRNA	GOLPH3, JUP, STAT6, CD206, E2F5, PFTK1:	Differentiation, Proliferation, Tumor Progression, Muscle Development,	^207,208^
5.	miR-183-5p	miRNA	FOXO1, SNAI2, ZEB1, E2F1, CTNNA2	Invasion, Angiogenesis, Cell Cycle Regulation, Cell Adhesion	^209,210^
6.	miR-186-5p	miRNA	AKT, VEGF, BCL2, ANXA9, XIAP, FGF2, RelA, TLR3, CALM2	Proliferation, Migration, Cell Signaling, Apoptosis, Angiogenesis, Ca2+ Regulation	^199,211^
7.	miR-20a-5p	miRNA	E2F1, PTEN, THBS1, Rab27B, IRF9, PPP6C	Cell Cycle, Apoptosis, Cell Proliferation	^212,213^
8.	miR-26b-5p	miRNA	CCND2, MCL1, TLR3, COL10A1, EZH2, COPS2, KPNA2, MRPL15, NOL12, PDE4B, CDK4	Diagnostic, Monitoring, Thyroid Carcinoma, Osteoarthritis, Immune Response, Cell Proliferation	^214,215^
9.	miR-30a-5p	miRNA	Beclin-1, ATG5, PTEN, SNAI1, CBFB, RRM2, AHNAK, DCBLD1	Diagnostic, Prognostic, Renal Cell Carcinoma, Mesothelioma, Cell Survival, Apoptosis, Cell Cycle Progression	^216,217^
10.	miR-143-3p	miRNA	KRAS, ERK5, Vimentin, CXCR4, SNAI1, CDH1, MYC, MMP-1	Diagnostic, Gastric Cancer, Cell Structure maintenance, Immune Response, Cell Adhesion, Apoptosis	^218,219^
11.	miR-96-5p	miRNA	FOXO1, PTEN, ZDHHC5, Aqp5, Celsr2, Myrip, Odf2, Ryk	Diagnostic, Acute Myocardial Infarction, Ovarian Cancer	^220^
12.	miR-122-5p	miRNA	CCNG1, ADAM17, SLC1A5, TP53, CCNG1, ADAM10, IGF1R	Diagnostic, Prognostic, Hepatocellular Carcinoma, AcuteMyocardial Infarction, Cell Cycle Regulation	^221^
13.	miR-302a	miRNA	Cyclin D1, E2F1, E2F7, AKT1, CDKN1A, CDKN1B, TGFBR2, RAB5C, GAB2, ERKs	Diagnostic, Germ Cell Tumors, Intracranial Germ Cell Tumors, Cell cycle Regulator, Cell Signaling Endocytosis,	^222,223^
14.	miR-501-3p	miRNA	MEF2D, TGFBR3, ACTR2, CDH1, COL1A1, RBBP5, RRM1, TPM3	Diagnostic, Alzheimer’s Disease, Synaptic Biomarker, Muscle Differentiation	^224,225^
15.	miR-144-3p	miRNA	ABCA1, FoxO1, Cyclin D1, CDK2, CDC25A	Diagnostic, Depression, Adipogenesis, Forensic Body Fluid Identification, Cholesterol Metabolism	^226,227^
16.	miR-302a-5p	miRNA	CDK2, CCND1, HMGA2, AKT1, CDKN1A, CDKN1B, TGFBR2	Diagnostic, Prognostic, Endometrial Carcinoma, Cell Cycle Control	^228^
17.	lncR_H19	lncRNA	Let-7, CaMKIIδ	Tumorigenesis, Apoptosis, Diagnostic Biomarker, Endometrial tolerance, Successful implantation of fetus	^229^
18.	lncR_ZEB2-AS1	lncRNA	ZEB2	Oncogenic, Tumorigenesis, Diagnostic Marker	^230^
19.	lncR_MALAT1	lncRNA	PGAM1, PGAM4, NOL6, NAP1L5, SESN1	Chemotherapy Resistance, Tumorigenesis, Prognostic Biomarker, Placental Implantation	^231^
20.	lncR_XIST	lncRNA	EZH2, CDK6	Prognostic Biomarker, Oncogene, Treatment Response	^232,233^

Human ectodermal stromal cell exosomes promote the development of endothelial tubes, a crucial step in the angiogenesis process, in addition to increasing trophoblast calmodulin synthesis, which improves invasive capacities. Mice experiments have shown that the administration of exosomes produced from embryonic stem cells can increase implantation rates and enhance the potential for implantation overall, resulting in improved blastocyst formation, embryo quality, and future development.^72^ Loaded with various proteins and nucleic acids, exosomes function as precise diagnostic indicators of pregnancy-related diseases. Examining exosomal lncRNAs can help identify biomarkers, provide a new basis for illness diagnosis and therapy, and shed light on the pathophysiology of several disorders linked to abnormal pregnancies. Numerous lncRNAs that control the activities of tumor cells may also have a major impact on trophoblasts, considering the parallels in proliferation, migration, and invasion between placental trophoblasts and tumor cells.^73^ This is especially noticeable in the pathways controlling cell cycle regulation, cellular invasion and migration, and angiogenesis.^74^ For example, lncRNA MALAT1 can suppress angiogenesis, cell cycle progression, apoptosis trophoblast development, migration, and invasion as well as recombinant hexokinase 2 (HK2) through its interaction with miR-216a-5p. Maternal peripheral blood can be used to identify PEXOs, which are rich in trophoblast-specific proteins, such as PLAP and HLA-G, and exosomal markers, such as CD9, CD63, and CD81. The concentration of PEXOs has the potential to be used as a predictor of fetal development and pregnancy viability. Changes in the nature and number of these exosomes might have a detrimental effect on target cells’ ability to function, according to histological investigations of these exosomes in a variety of illnesses.^75^

## 4 Role of Exosomes: Preeclampsia

Preeclampsia (PE), a severe and prevalent condition that may be referred to as “Gestohypertoxemia,” is defined as new-onset gestational hypertension after 20 weeks of pregnancy in tandem with proteinuria (creatinine ratio ≥30 mg/mmol, ≥300 mg/24 h, or ≥2+ on dipstick)^11^ high blood pressure (160/110 mmHg) and end-organ-damage that possess the risk of mortality and morbidity for both mother and the fetus.^76,77^ In 2024 alone, PE is a stealthy predator, causing 76,000 maternal and 500,000 neonatal deaths annually, especially in healthcare-scarce regions. Affecting about 2–10% of pregnancies, its prevalence has steadily increased over the past 30 years. The American Heart Association (AHA) has issued guidelines recognizing a history of PE as a distinct factor for cardiovascular disease.^78^ PE beginning in early pregnancy is characterized by reduced placental perfusion due to impaired extravillous trophoblast invasion and inadequate spiral artery remodeling, leading to shallow placentation and diminished perfusion. To allow for a more precise prognosis and prevention of this disorder, a thorough evaluation of the etiology and pathogenesis of PE is necessary. The condition involves early chronic inflammation, with leukocyte activation and high cytokine levels. Tumor necrosis factor-α (TNF-α) is a multifunctional cytokine that triggers vascular dysfunction through disturbances in angiogenesis by enhancing the expression of intercellular adhesion molecule-1 (ICAM-1) on endothelial cells and trophoblasts, contributing to PE. These factors lead to poor angiogenesis, reduced placental growth factor (PlGF), and uteroplacental retardation, ultimately resulting in PE.^79,80^ The severity of preeclampsia varies from mild to severe and can lead to complications such as Eclampsia and the “HELLP” syndrome^81^ (hemolysis, increased liver enzymes, and low platelet count). Exosomes play a cornerstone role in preeclampsia, as placenta-derived exosomes released from syncytiotrophoblast cells into the perinatal systemic milieu exhibit fluctuating levels in patients with preeclampsia compared to those with normal pregnancies i.e., 1.47-fold and 1.45-fold higher.^82,83^ Due to elevated levels of sFlt-1 (soluble fms-like tyrokinase-1) and sEng (soluble Endoglin), which are linked to vascular dysfunction, exosomes may affect distant organs through immune modulation and extracellular matrix formation.^84^ Reports indicate that higher concentrations of exosomes are observed in preeclampsia,^85^ suggesting their potential as biomarkers for this intricate pathophysiological condition. These variations could be utilized for early diagnosis of preeclampsia.^38^ In addition, Ermini et al. (2017) stated that the exosomes deriving from PE were potentially implicated in promoting vascular dysfunction due to their high content of sFlt-1 and sEng.^86^ Increased levels of sFlt-1 and sEng contained in exosomes are responsible for impaired vascular angiogenesis and also contribute to the pathogenesis of preeclampsia since they deplete the PlGF level for reduced angiogenesis during placentation.^84,87^ In PE patients, the elevated concentrations of circulating sFlt-1 interact with the VEGF-1 receptors on the endothelial cells, thereby interfering with endothelial cell communication, leading to vascular dysfunction, hypertension and decreased levels of VEGF and PlGF.^88^

MicroRNAs such as miR-210, miR-155, and miR-29b represent the hypoxia-induced miRNA group, also referred to as hypoxamiRs. These appear to offer great promise as biomarkers for monitoring pregnancies affected by preeclampsia. The role of miR-210 seems to be of utmost significance for cell proliferation in response to DNA damage, mitochondrial oxidative metabolism, and angiogenesis.^89,90^ Enhanced levels of miR-210 inhibited cell migration trophoblast invasion,^91^ plus its elevated levels are found in preeclampsia patients.^92^ Hypoxic induction of miR-155 in HUVECs (human umbilical vein endothelial cells) makes it challenging to regulate angiogenic responses.^93^ Whereas miR-155 is said to be negatively regulated by the overexpression of LNC00240 (Long intergenic nonprotein coding RNA 240) on trophoblasts in preeclampsia, which inhibits the oxidative stress-induced pyroptosis by silencing the miR-155.^94^ Histone deacetylase 4 (HDAC4) modulates the motility of trophoblast cells, and this modulation can actually be counteracted by miR-29b^95^ since it induces apoptosis and inhibits invasion and angiogenesis of trophoblast cells.^96^ A recent study reported that syncytiotrophoblast-derived extracellular vesicles from preeclampsia placentae (preeclampsia-STBEVs) stimulate LOX-1, which causes endothelial dysfunction. Preeclampsia-STBEVs lowered nitric oxide’s role in relaxation, which TS20 prevented. Superoxide dismutase or apocynin, an inhibitor of NOX (nicotinamide adenine dinucleotide phosphate oxidase), reversed decreased endothelial-dependent vasodilation in arteries exposed to preeclampsia-STBEVs^97^ (Figure 3). The exploration of exosomes unveils a new frontier in the fight against preeclampsia. These nanoscale messengers, with their capacity to influence immune responses and vascular dynamics, present a novel biomarker for early detection. Embracing the potential of exosomes could lead to groundbreaking advancements in therapeutic interventions, paving the way for healthier pregnancies and improved maternal–fetal outcomes in this intricate gestational disorder.

**Figure 3:**
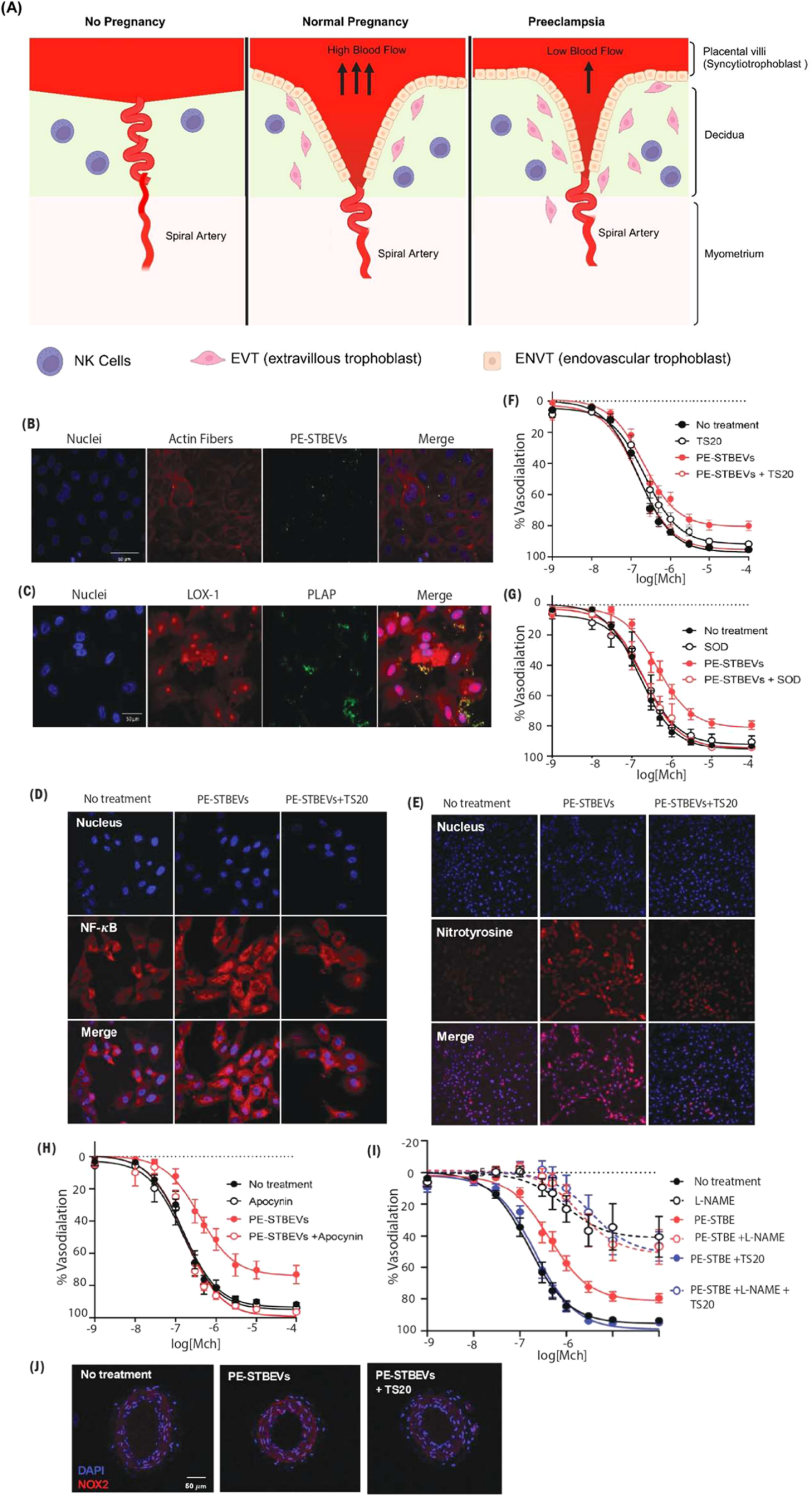
Placenta-derived extracellular vesicles from preeclamptic pregnancies impair vascular endothelial function via lectin-like oxidized LDL receptor-1. (A) Schematic representation of the comparative normal pregnancy vs preeclampsia condition in pregnancy (created with Biorender.com), (B) confocal microscopy images showing the uptake and internal localization of STBEVs in (80 μg/mL) for 4 h, (C) immunofluorescence and confocal microscopy images for LOX-1 and PLAP STBEV expression, (D) NF-κB location visualized by immunofluorescence and confocal microscopy in HUVECs treated with or without PE-STBEVs in the absence or presence of TS20 (LOX-1 inhibitor), (E) confocal microscopy images for nitrotyrosine (indicative of nitrative stress in HUVECs) treated with PE-STBEVs in the absence or the presence of TS20, (F–I) concentration response curve of methylcholine (MCh)-induced vasodilation in preconstricted mesenteric arteries incubated overnight with or without PE-STBEVs and with or without TS20, SOD, apocynin, and L-NAME (N­[G]-nitro-l-arginine methyl ester), (J) confocal microscopy of NOX2 (nicotinamide adenine dinucleotide phosphate oxidase 2) in mesenteric arteries incubated overnight with or without PE-STBEVs. (Reproduced with permission from ref 97. Copyright @2023 American Heart Association/American Stroke Association Journals.)

## 5 Role of Exosomes: Gestational Diabetes Mellitus

Gestational diabetes mellitus (GDM) represents impaired glucose tolerance, which occurs for the first time during pregnancy. Its incidence correlates with the rising prevalence of obesity and type 2 diabetes mellitus (T2DM).^10^ Women diagnosed with GDM face an increased risk of developing hypertension, potentially leading to complications such as preeclampsia or Eclampsia during pregnancy.^98^ GDM is considered to have multiple contributing factors, although its etiology remains unclear. A recent umbrella review encompassing 30 meta-analyses identified 61 potential risk factors, highlighting common associations such as overweight or obesity, family history of diabetes, hypothyroidism, sleep-disordered breathing, and polycystic ovary syndrome.^99^ GDM can strike at any point during pregnancy, typically between 24 and 28 weeks. Early detection and management are crucial because GDM can have adverse short- and long-term consequences for both the mother and baby. Unfortunately, there’s also a high chance (up to 48%) of GDM recurring in a future pregnancy.^100^ Unraveling the global prevalence of GDM gets tricky due to differing standards for diagnosis across regions. A recent large-scale study by Saeedi et al. 2021 estimated that 14.7% of pregnancies globally are affected by GDM.^101^ In 2019, a meta-analysis that used the same diagnostic criteria revealed a significant geographic disparity in GDM rates. The highest pooled prevalence, at 11.4%, was observed in South Asia (Bangladesh, India, and Sri Lanka), compared to a much lower range of 3.6–6.0% in other parts of the world.^102^ The pathogenesis of GDM is complex and is not fully understood. Research suggests a combination of factors might be at play, including insulin resistance, inflammation, oxidative stress, adipose tissue, and endothelial cell dysfunction.^103^

Studies are uncovering the potential of EVs to shed new light on the mechanisms behind them. These membrane-encapsulated particles are released by cells into their extracellular environment under both physiological and stress conditions, including injury or cell death.^104,105^ Extracellular vesicles (EVs) are present in several biological fluids including blood, cerebrospinal fluid, tears, urine, and ascites. During pregnancy, EVs originating from the placenta can be detected in the mother’s bloodstream as early as 6 weeks into gestation, and their levels rise as the pregnancy advances.^34^ In women with GDM, the total number of exosomes in maternal plasma between 11 and 14 weeks of gestation is up to two times greater compared to non-GDM pregnancies.^106^ Exosomes derived from the placenta participate in maternal changes of islets maladaptation during GDM., which are significantly promotes β cell apoptosis, impairs the GSIS *in vitro*, and directly causes impaired glucose intolerance in pregnant mice^107^ (Figure 4). Exosomes released from trophoblasts in GDM patients induce the secretion of proinflammatory cytokines (IL-18, IL-1β) and promote the proliferation, migration, and tube formation of umbilical vein endothelial cells.^108^ Additionally, in diabetic patients, microRNA-326 is upregulated, which negatively correlates with its target, adiponectin, potentially mediating the inflammatory responses typically associated with GDM. However, the results regarding several microRNA candidates as biomarkers for GDM are often inconsistent.^109^ Changes in EV biogenesis are frequently found in cardiovascular diseases, including diabetes, and can be measured in altered amounts in various biofluids. Depending on the source of the EVs investigated, such as plasma, urine, or other biofluids, these variations may reflect diabetes consequences such as endothelial injury, kidney damage, or, in the setting of pregnancy, placental stress.^28^ A study by James-Allan and co-workers found that pregnant women had significantly higher levels of EVs in their blood compared to nonpregnant women.^110^ Interestingly, these levels were even higher in pregnant women with gestational diabetes mellitus (GDM). This suggests that the placenta might be releasing more EVs in pregnancies with GDM. In recent years, interest in the research on exosomes during pregnancy has been growing. A prospective study found that visceral fat thickness might predict GDM by regulating the miRNA-148 family of adipose-derived exosomes. A study conducted on obese mice found that their exosomes were enhanced with microRNAs, which caused glucose intolerance and insulin resistance in lean mice. This effect was initially attributed to the role of adipose-derived exosomal miRNAs in metabolic dysregulation.^111^ Further research has demonstrated that visceral adipose tissue exosomes modulate miRNA-148a and miRNA-148b expression, leading to impaired insulin signaling.^112–114^ These findings suggest that dysregulated miRNA expression in adipose-derived exosomes, driven by visceral fat accumulation, may play an important role in the pathogenesis of GDM. However, there are a panel of miRNA that has been studied extensively in recent years which are studied by Liu, et al. (2021), who found a significant upregulation of miR-98 derived from the placenta at a gestation of 37–40 weeks in GDM (*n* = 193) compared to normoglycemic pregnancies (*n* = 202), suggesting its involvement in insulin resistance.^115^ A recent study demonstrated a significant reduction in miRNA expression in GDM. Specifically, miRNA-148a levels were reduced by 45% in the normal-weight (NW) GDM group and by 61% in the overweight/obese (OW/OB) GDM group compared with the NW non-GDM group. Similarly, miRNA-30b levels were 65% lower in the NW GDM group and 64% lower in the OW/OB GDM group relative to the NW non-GDM group. These findings emphasized the critical need for integrating quantitative analyses to elucidate the diagnostic and prognostic potential of exosomal miRNAs in GDM.^116^ As well as Chen et al. 2022 reported a significantly higher concentration of placenta-derived exosomes in GDM patients (∼2.2-fold, ∼1.5-fold, and ∼1.8-fold increase at respective gestational ages) compared to normal pregnancies, demonstrating that, maternal hyperglycemia enhances placental exosome release into circulation.^117^ Further investigation into the mechanisms of exosomes in both normal pregnancy and GDM will enhance our understanding of the function of circulating exosomes in patients with GDM and the pathophysiological mechanisms underlying GDM. This knowledge could provide a basis for improving pregnancy outcomes by enabling the development of better diagnostic and therapeutic strategies.

**Figure 4:**
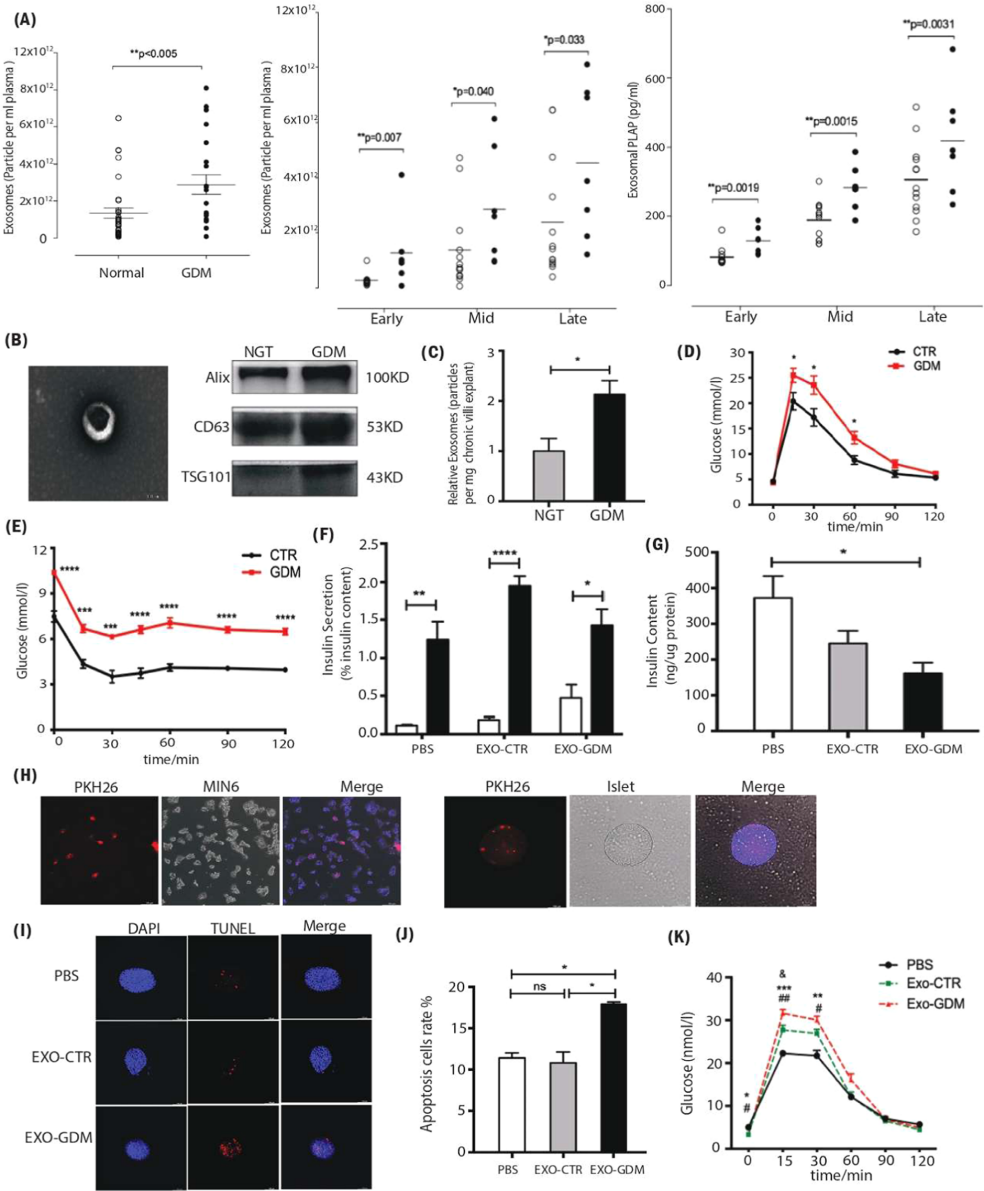
Role of exosomes in gestational diabetes. (A) Total exosome number presented as the average across early, mid, and late gestation (reproduced with permission from ref 106. Copyright @2016 American diabetes association). (B) Visualization of the exosomes released from chorionic villi explants by electron microscopy and representative exosome marker Alix, CD63, and TSG101 were measured by Western blot analysis, (C) Concentration of exosomes from normal and GDM pregnancies, (D) glucose tolerance test (GTT), (E) insulin tolerance test (ITT) performed on a cohort of high-fat (GDM) diet- and control (CTR)-fed pregnant dams at gestational day 12.5, (F) glucose-stimulated insulin secretion (GSIS), (G) insulin content during GSIS tests, (H) mice placenta-derived exosomes uptake in MIN6 cells and islets after 24 h of coculture, (I) fluorescence microscopy of treated islets stained with TUNEL signals (red) and DAPI (nucleus, blue), (J) apoptosis was measured by the 7AAD-Annexin V test and presented by the histogram graph, (K) glucose tolerance test (GTT) on control pregnant recipient mice after adoptive transfer of PBS, Exo-CTR, and Exo-GDM mice at GD16.5. (Reproduced from ref 107. Available under a CC-BY 4.0 license. Copyright 2024 The Authors. Published by Frontiers on behalf of Fronteirs of Endocrinology.)

## 6 Role of Exosomes in Pregnancy Loss and Preterm Birth

The first trimester, which marks the early stages of pregnancy, is a critical period for expectant mothers, as pregnancy loss (PL) during this time is the most common complication requiring attention. Recent statistics reveal a heartbreaking figure, in which 23% of cases reported specifically in the first trimester (≤12 weeks) are attributable to miscarriage, whereas the scenario is equally alarming, with 42.39 million cases of pregnancy loss reported worldwide.^118^ PL is a routinely encountered complication, affecting about 15% of apparently healthy couples.^119^ Recurrent pregnancy loss (RPL) is defined as three or more consecutive losses before 12 weeks of gestation, although some guidelines require only two cases for categorizing the condition to be RPL. RPL affects 1–3% of all couples. This high prevalence is primarily attributed to genetic abnormalities, endocrinological issues, thrombophilic autoimmune and alloimmune disorders, and uterine abnormalities. Thereby, the said conditions are treated as severe risk factors for RPL.^120,121^ The mechanisms involved in RPL pathogenesis include insufficient trophoblast invasion, villitis, and placental vessel microthrombi.^122^ The key concern is not limited to a single occurrence; patients with recurrent pregnancy loss (RPL) face a higher risk of complications in subsequent pregnancies, as well. Therefore, accurate diagnostic tools for predicting RPL have become a necessity not only for diagnosis but also for a basic understanding of the syndrome. Study shows that preterm birth occurred in mice treated with late gestation-derived exosome-treated mice, which increased inflammatory mediators in the cervix, uterus, and fetal membranes but not in the placenta, which was not observed in mice injected with early gestation exosomes. This suggests that exosomes function as paracrine mediators of labor and delivery^123^ (Figure 5). In modern healthcare settings, there has been an emerging interest in the diagnostic potential of EVs. These small plasma membrane vesicles are essential for intercellular communication and are implicated in various pathological processes. EVs perform numerous functions, including critical roles within the immune system, and they condense and protect their cargo within the vesicles.^124^ Emerging evidence increasingly suggests that increased placental oxidative stress plays a significant role in the pathogenesis of early pregnancy loss, primarily by delaying trophoblast invasion.^125^ The elevation in oxidative stress is known to induce DNA damage within the placental tissue.^126^ Research on exosomal DNA damage in recurrent pregnancy loss (RPL) is expanding, with studies indicating that abnormal DNA methylation may contribute to RPL by influencing implantation, fetal growth, and development. The abnormal DNA methylation of imprinted, placenta-specific, immune-related genes and sperm DNA may influence embryo implantation, growth, and development, ultimately contributing to RPL.^127^ Furthermore, it disrupts the protein-folding mechanism, leading to an augmented production of misfolded proteins.^128^ An elevated level of cellular senescence is frequently associated with various pregnancy complications, including preeclampsia^126^ and miscarriages.^129^ Recent studies have also identified exosomal microRNAs as key regulators of oxidative stress responses in the placenta, highlighting their potential as biomarkers for early pregnancy loss.One of the mechanisms by which exosomes regulate trophoblast invasion is through the transfer of miRNAs that influence trophoblast migration and proliferation. The miR-486-5p affects the activity of the trophoblast. In this study, the author found that this exosomal miR-486-5p influences trophoblast cell function by targeting the IGF1 signaling pathway, thus controlling proliferation and invasion.^130^ Another study highlighted the mechanism by which MSC-derived exosomes regulate trophoblast invasion. According to this study, MSC-derived exosomes, carrying high levels of H19, promote trophoblast invasion and migration and suppress apoptosis by decreasing let-7b, increasing FOXO1, and activating the AKT pathway.^131^ According to some previous study findings, the present report demonstrated that in cases of miscarriage proteins linked to senescence, DNA damage, and endoplasmic reticulum (ER) stress are not released via EVs as typically expected. Instead, these proteins accumulate within placental tissue. Recent investigations have clarified that placental EVs derived from complicated pregnancies can significantly impact the function of target cells by releasing their cargo, which includes harmful proteins such as misfolded proteins and Mixed Lineage Kinase domain-like (MLKL) proteins.^130^ It is evident that misfolded proteins are associated with PE. These proteins, including amyloid β-peptide, α-1 antitrypsin, albumin, IgG k-free light chains, and ceruloplasmin, are dysregulated in PE, leading to the deposition of amyloid-like aggregates in the placenta and body fluids.^132^ This accumulation of proteins induces ER stress, activating the unfolded protein response (UPR) to promote ER-associated degradation and maintain ER homeostasis.^133^ A study conducted by Zhang and researchers revealed that elevated levels of three senescence-repair-associated proteins (RPA-70, PMSE-4, and PAK-2) were observed in placental EVs. However, these proteins were found at lower levels in placental tissue from missed miscarriages. Interestingly, when EV formation or release was inhibited using GW4869, the expression levels of these three proteins increased in GW4869-treated placental explants from missed miscarriage cases.^28^ This suggests that the “inadvertent” sorting and export of senescence-repair-associated proteins by the EVs secreted from the placenta may be linked to the abnormal functioning in the development of the placenta observed in miscarriages. Thus, placental EVs play a crucial role in modulating placenta function and may have significant implications for understanding and diagnosing pregnancy complications.

**Figure 5:**
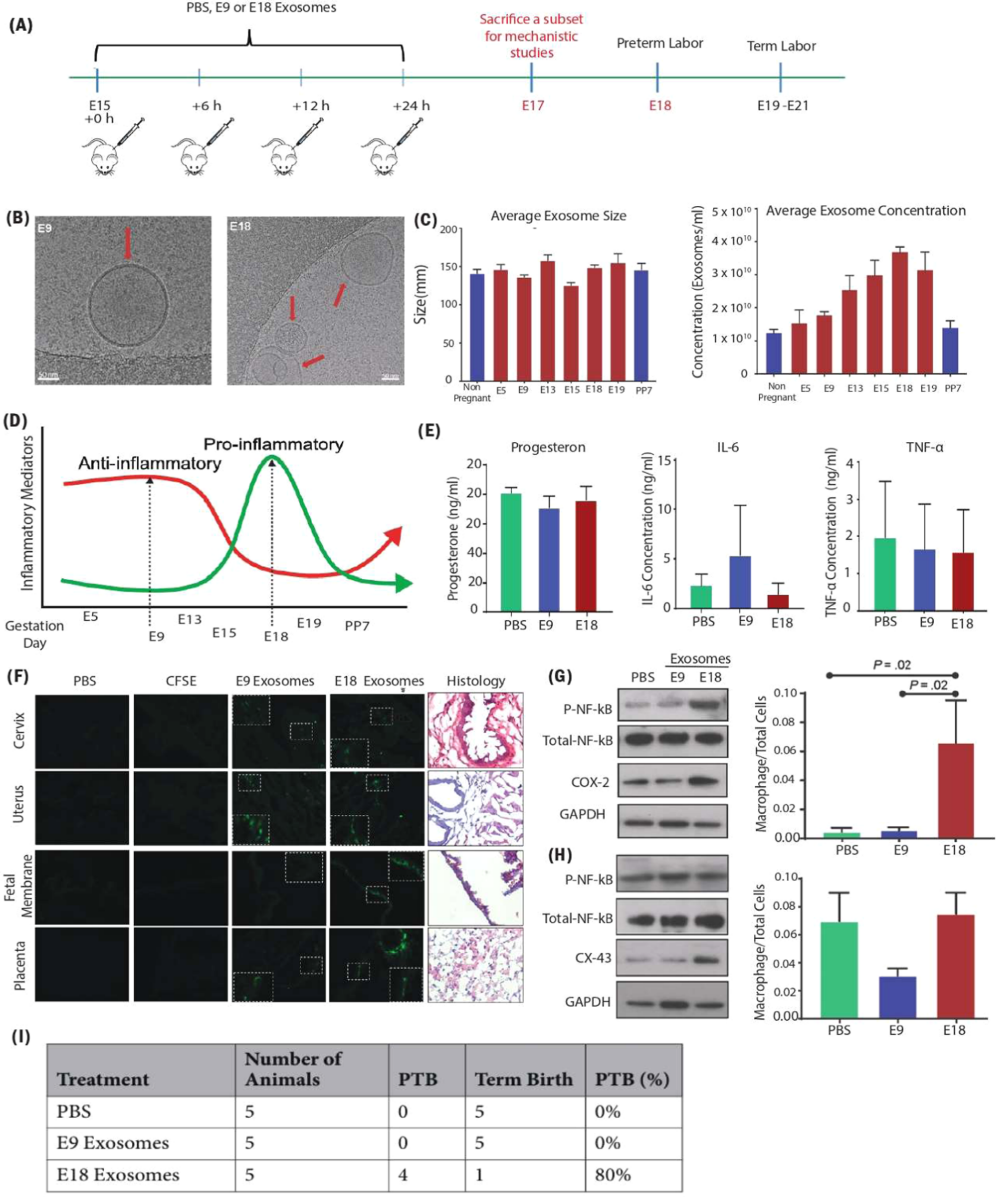
Role of Exosomes in preterm birth. (A) Experimental design for PBS and exosome injections to determine the functional role of early (E9), and late gestation (E18) exosomes *in vivo*, (B, C) a representative cryo-electron microscopy image and NTA analysis of exosome from early gestation (E9) and late gestation (E18), (D) graphical representation of inflammatory changes during mouse pregnancy, also reflected in exosomes throughout gestation, (E) Bio plex analysis of maternal plasma progesterone, IL-6, and TNF-α in PBS, E9, and E18-injected mice, (F) fluorescently labeled exosomes injected into pregnant mice traffic to the cervix, uterus, fetal membranes, and placenta and representative H&E staining for orientation and localization, (G, H) exosomes injected into pregnant mice induce labor-associated changes in the cervix and uterus evaluate by western blot analysis and densitometry quantitation, and (I) preterm birth rates in PBS, E9, and E18 exosome-injected mice. (Reproduced from ref 123. Available under a CC-BY 4.0 license. Copyright 2019 The Authors. Published by Nature on behalf of Scientific Reports).

## 7 Immunomodulatory Role of Exosomes in Pregnancy

Cellular communication, particularly in higher life forms such as mammals, by and large, remains one of the most intricate physiological processes at the molecular scale. Exosomes are secreted by diverse cell types in both prokaryotes and eukaryotes,^36^ encompassing immune cells like B and T-lymphocytes and associated signaling mechanisms in humans, and play a pivotal role in gestational dynamics.^134^ These pathways have been extensively investigated in recent years, as they are prime drivers for vesicle secretion at all times for the information exchange among the cells to maintain homeostasis. Recent literature has revealed novel exosome signaling pathways in immune tolerance during pregnancy, emphasizing the crucial role of extracellular vesicles (EVs) in maternal–fetal immune modulation, thus, significantly advancing our understanding. Exosomes secreted by trophoblasts and immune cells have been shown to carry essential proteins, RNAs, and miRNAs that regulate maternal immune adaptation. As an illustration, IL-35-expressing exosomes have been characterized as a crucial element in preventing maternal immune rejection of the fetus by modulating T-cell responses.^135^ In the state of pregnancy, it becomes inevitable for the maternal immune system to develop tolerance toward the semi/fully allogeneic fetus and overcome immune intervention through suppression mechanisms to support the maternal adaptation to pregnancy.^136^ Mechanisms, like the β-catenin and PI3K/Akt signaling pathways activated by mesenchymal stem cell-derived exosomes (MSC-Exos), have been found to promote vascular remodeling and immune tolerance, offering new therapeutic prospects for pregnancy-related disorders.^137^ Exosomes are crucial players as they facilitate fetal–maternal communication and thus significantly reduce the possibilities for fetal rejection.^138^ The placenta is established between the developing fetus and the mother around 5–6 days after conception. It stretches up to 20 cm in length and 3 cm in thickness^139^ and performs all-round functions viz immune, endocrine, respiration, circulation, and nutrition, thus becoming responsible for the definitive growth of the fetus.^140^ The placenta is also a generous source of secretion of exosomes that carry immunomodulatory molecules to protect the fetus against maternal immune attack. Placental trophoblast cells from the inner lining of the fetal side of the placenta and secret exosomes that carry cargo molecules like Human Leukocyte Antigen-G (HLA)-G molecules,^141^ cytokines and chemokines, MHC molecules, micro and noncoding RNAs, Fas ligand (FasL),^142^ galantines, etc. that modulates subpopulations of the maternal immune cells like B and T lymphocytes and aids in regulating the proliferation of these cells.^143^

Fetal endothelial cells are observed as early as the third and fourth week of gestation and are involved in the development of vasculature in the fetus.^50^ These cells release exosomes containing angiogenic factors viz., Vascular Endothelial Growth Factor (VEGF) and Placental Growth Factor (PlGF) that bind to the receptors of the abundant uterine NK cells, thus triggering the production of anti-inflammatory cytokines, which influences the formation of more immune tolerant space for the fetal growth.^144^ The outer layer of the placenta is lined by the syncytiotrophoblast cells that are observed during the sixth–eighth week of gestation, secreting specialized extracellular vesicles. Syncytiotrophoblast-derived exosomes carry immunosuppressive biomolecules such as lipids and nucleic acids that act upon maternal dendritic cells and NK cells to suppress their activity. They also aid in maintaining immune tolerance at the maternal–fetal interface by suppressing inflammatory responses.^145^ Another study illustrated that the STAT1/IRF1 pathway is a crucial regulator in exosome-mediated immune tolerance, particularly through the polarization of decidual macrophages.^146^ In addition to that, they release cytokine regulatory factors to monitor the levels of proinflammatory ones (TNF-α) to cease the probability of future complications^147^ and special ligands like TNF-related apoptosis-inducing ligand (TRAIL), which is an immunosuppressive agent and induces apoptosis in active PBMCs.^28^ Similarly, the other cells of the placenta, like fetal hematopoietic and fetal epithelial cells,^13^ also secrete exosomes pool, potentially contributing to the Immunomodulation of maternal innate and adaptive immune cells.

Exosomal microRNAs (miRNAs) from placental origin are crucial elements for the regulation of maternal–fetal immune tolerance, modulating innate and adaptive immune responses to preserve pregnancy. The mechanistic background of their functional dynamics shares significant parallels with immune evasion strategies observed in cancer and infectious diseases. The placenta actively releases extracellular vesicles (EVs) rich in miRNAs, encompassing members of the chromosome 19 microRNA cluster (C19MC), which aid in maternal immune activation suppression, thus promoting immune tolerance.^148^ These exosomal miRNAs target antigen-presenting cells (APCs), lowering the major histocompatibility complex (MHC) class II and costimulatory molecules (CD80/CD86) expression, thereby compromising dendritic cell (DC) function and antigen presentation.^149^ T cells (Tregs) are instigated by placental exosomal miRNAs viz., miR-517a and miR-146a by upregulating transforming growth factor-β (TGF-β) and interleukin-10 (IL-10), transforming maternal immunity toward an anti-inflammatory state.^150^ Along with that, miR-378a and miR-29a downregulate the natural killer (NK) cell activation receptor NKG2D, downregulating NK cytotoxicity against trophoblast cells and warranting fetal survival.^151^ Another critical mechanism involves miRNA-mediated upregulation of programmed death ligand 1 (PD-L1), which advocates maternal CD8+ T-cell exhaustion, a strategy mirroring immune checkpoint activation in tumors.^152^ Such immune-modulatory processes of placental exosomal miRNAs are not specific to pregnancy but are also extrapolated by cancer cells and infectious pathogens for immune evasion. miRNAs e.g., miR-105 and miR-200, etc are packaged into tumor-derived exosomes in case of cancer which advances epithelial-to-mesenchymal transition (EMT) while also enhancing PD-L1 expression, resulting in T-cell dysfunction and immune escape.^153^ Tumors also utilize miRNAs such as miR-21 and miR-146a to suppress IFN-γ production, reducing CD8+ T-cell cytotoxicity, thus systematically imitating placental immune tolerance mechanisms.^154^ Similarly, viral and bacterial pathogens also exploit exosomal miRNAs to evade immune surveillance. For instance, viral miRNAs are incorporated into exosomes by Epstein–Barr virus (EBV) and human immunodeficiency virus (HIV) to subdue type I interferon responses, inhibiting JAK/STAT signaling, and blockage of antigen presentation.^155^ The placenta’s capability to modulate immune tolerance via exosomal miRNAs suggests an evolutionary modification that tumors and pathogens utilize to evade immune clearance.

Immune reprogramming of maternal monocytes, dendritic cells (DCs), and NK cells is yet another attribute of exosomes in the gestational landscape. Fang et al., 2024 suggest that the placenta-derived exosomes carrying miR-29a-3p suppress decidual NK cell IF-Y production, promoting immune tolerance but dysregulation in unexplained recurrent pregnancy loss (uRPL) patients, leading to altered NK cytotoxicity.^156^ The reprogramming of maternal monocytes via exosomal miRNAs (e.g., miR-410-5p) induces M2 macrophage polarization by means of STAT1 inhibition, which is disintegrated in preeclampsia and fetal growth restriction^157^ (IUGR). Trophoblastic exosomes with HLA-G influence monocyte-derived DC differentiation, transferring them toward tolerogenic DCs, indispensable for maternal immune adaptation^144^ (Mincheva-Nilsson, 2024). Aberrations in exosomal HLA-G cargo correspond with gestational diabetes mellitus (GDM) and preterm birth.^39^ Placental exosomes also govern NK cell activation via TGF-β1 and galectin-1 cargo, which leads to decreased secretion of granzyme B, which is impaired in preeclampsia, resulting in amplified maternal NK cytotoxicity and trophoblast apoptosis.^158^ The role of exosomes in maternal pregnancy tolerance is so extensive that apart from the placenta-associated cells of the fetus, a myriad of extracellular vesicles are being released from the maternal half as well, viz., exosomes from Myeloid-derived suppressor Cells (MDSCs) from the bone marrow of pregnant women impacting T-cell responses,^159^ Progesterone-Induced Blocking Factor (PIBF), a speculated cargo of EV which is a progesterone hormone induced protein,^160^ Multipotent Mesenchymal Stromal Cells^161^ (MSCs), etc., strongly suggests the significance of extracellular vesicles role to serve the purpose of immune modulation in pregnancy. The recent study reported that first-trimester placenta-derived exosomes (pEXOs) contribute to regulating maternal immune tolerance by reprogramming the circulating monocytes^51^ (Figure 6). Exosomal engineering is an upcoming novel paradigm that involves either exogenous or endogenous modifications in the natural exosomes by manipulating their membrane or cargo molecules for precisely targeted therapeutics, which re-emphasizes the therapeutic potential of exosomes in immune modulation during pregnancy complications. Recent progress in this research avenue has given rise to Placental Exosome-Laden Artificial Xenogenic Organelles (PlaXosomes), which imitate intrinsic placental exosomes, thus lowering the secretion of proinflammatory cytokines such as TNF-α and IL-6 while promoting T-regulatory cell expansion and hence reducing the risk of preeclampsia.^162^ CRISPR/dCas9-modified exosomes have been engineered for the epigenetic repression of implantation failure-associated genes leading to increased blastocyst adhesion rates by 37% in preclinical models.^163^ These advanced approaches highlight the therapeutic precision of exosome-based interventions in pregnancy disorders.

**Figure 6:**
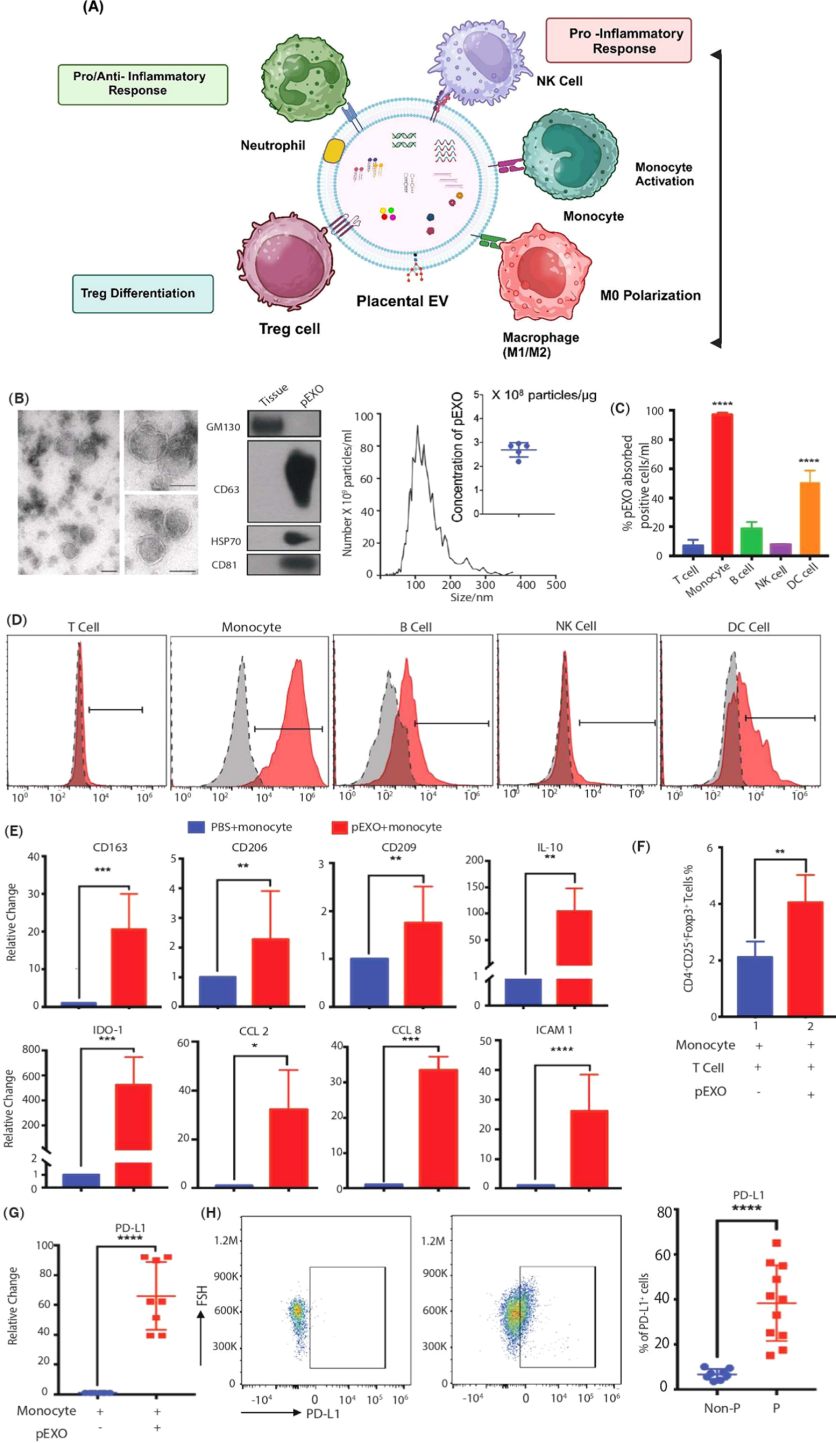
Immunomodulatory effect of placenta-derived exosomes on different immune cells. (A) Schematic representation of immunomodulatory effect of placenta-derived exosomes on different immune cells (created with Biorender.com), (B) TEM images, NTA analysis, and western blot exosome markers CD63, HSP70, CD81, and GM130 (Golgi marker) of pEXO, (C) percentage of carboxy-fluorescein succinimidyl ester (CFSE)-labeled pEXO positive cells, (D) flow cytometric analysis interaction of pEXO with CD14+ monocytes, B cells and dendritic cells have a mild interaction with pEXO, while T cells and NK cells have barely any interaction, (E) M2 macrophage markers: CD163, CD206, CD209, IL-10, CCL-2, CCL-8, IDO-1, and HLA-DRA of pEXO-polarized and control macrophages determined by RT-QPCR, (F) flow cytometric analysis showing the increased population of Treg cells (CD4+CD25+Foxp3+) in coculture of autologous T cells and pEXO-educated monocytes, (G) enhanced expression of PD-L1 at the mRNA level in the pEXO-educ monocyte (*n* = 8), and (H) increased frequencies of CD14+PD-L1+ monocytes from pregnant women and nonpregnant control (Reproduced from ref 51. Available under a CC-BY 4.0 license. Copyright 2022 The Authors. Published by BioMed Central on behalf of the Journal of Nanobiotechnology.).

## 8 Exosome-Associated Biomarkers for Pregnancy Complications

In recent years, pregnancy-associated complications like intrauterine growth restriction, placental abruption, preterm and stillbirths, GDM, preeclampsia, etc., have been very evident. The increased spikes in the rates of mortality and morbidity among pregnant women thus significantly affecting the health of mother and child. There are numerous underlying reasons, such as pre-existing conditions like high blood pressure leading to chronic hypertension^164^ (AOCG—Chronic hypertension in pregnancy), autoimmune disorders, age, weight, conditions of multiple gestations (ACOG—Obstetric Care Consensus), diet, stress, infections and other poor lifestyle choices of the mother, etc. These factors exert a consequential influence on exosome dynamics, attuning both the quantity and bioactive cargo of extracellular vesicles (EVs) secreted into maternal and fetal ecosystems. Multiple studies have illustrated how maternal dietary integrants, specifically high-fat or processed diets, remodel the composition of breast milk exosomes, affecting miRNA profiles that govern immune development, metabolic programming, as well as neural development in infants.^165,166^ Chronic maternal stress has been shown to effect the release of exosomes through glucocorticoid-mediated modulation of nSMase2 activity, giving rise to modifications in miRNA cargo, with indications for inflammatory responses and fetal vulnerability to asthma and obesity.^53,167^ Congenital infections due to viral and bacterial exposures have been reported to alter exosome secretion rates and supplement exosomes with pathogen-derived nucleic acids and proteins, potentially leading to neonatal immune priming and escalating risk for long-term metabolic and inflammatory diseases.^168^ Furthermore, maternal habitat is also a key factor for fetal development as studies have revealed that environmental pollutants and associated oxidative stress can trigger exosomal release possessing stress-responsive miRNAs and proteins, further aiding to fetal programming of chronic diseases.^169^ In conclusion, all of these findings emphasize that maternal diet, psychological stress, and infections are not just transient conditions but critical modulators of exosomal signaling pathways that may imprint lifelong health trajectories in the offspring. The epigenetic and immunomodulatory effects mediated through exosome alterations highlight the need for tailored maternal care strategies during pregnancy and lactation.^170,171^

During normal pregnancy, the rate of exosomal secretion increases drastically, peaking in the third trimester, which suggests maximal placental activity and fetomaternal communication.^172^ In preeclampsia (PE), exosome concentrations are significantly elevated as early as the second trimester, with modifications in cargo containing increased antiangiogenic factors like sFlt-1 and inflammatory mediators like TNF-α and IL-6.^173,174^ The exosomal proteome in PE is enriched with proteins that play crucial roles in oxidative stress and endothelial dysfunction, diverging from the principal antioxidant and vascular regulatory proteins in normal pregnancies.^175^ Exosomes carrying altered adipokines and glucose transport regulators, like downregulated GLUT4 and increased resistin, are observed in GDM, correlating with maternal insulin resistance.^176^ At the miRNA level, PE-exosomes show increased miR-210 and miR-136, resulting in dysregulated angiogenesis and trophoblast invasion, whereas GDM exosomes display elevated miR-29a and miR-330-3p, both linked to β-cell dysfunction.^177,178^ Comparative studies show the shift in exosomal lipid compositions from phosphatidylserine-rich profiles in normal pregnancies to ceramide-enriched patterns in PE, denoting remodeled pathways of vesicle biogenesis.^179^ Placental extracellular vesicles from severe PE patients dysregulate cardiomyocyte calcium balance in vitro, indicting systemic off-target effects.^180^ Furthermore, endometrial EV secretion rates and cargo are hormonally regulated in early gestation, and aberrations therein are associated with early onset PE and GDM.^107,177^

Exosomes have been emerging as remarkable biomarkers for the noninvasive diagnosis of pregnancy complications. Based on the specific types of cells/tissue that secrete them and the cargo molecules present, they are considered unique biomarkers in liquid biopsy assessment for early disease diagnosis and prophylaxis^34^ concerning disorders like preeclampsia and GDM discussed in Table 2. Isolation of exosomes from clinical samples is a crucial component in extrapolating their efficacy for diagnostic, therapeutic, and basic biomedical research. A vertical of exosome research is devoted to improving the existing techniques by consolidating modern hybrid methods to optimize purity, yield, and efficacy. Conventional approaches such as ultracentrifugation (UC), size-exclusion chromatography (SEC), and immuno-affinity capture (IAC) have been extensively used, yet each method has its limitations. By examining recent research holistically, we can appreciate how these techniques complement each other rather than exist as isolated methodologies.

**Table 2:** Comprehensive List of Potential Biomarkers for Early Diagnosis of Preeclampsia and Gestational Diabetes Mellitus

sr. no	biomarker for preeclampsia	nature	source	isolation/detection/quantification methods	function/potential role	references
	**For Preeclampsia**					
1.	Placental Growth Factor (PlGF)	Pro-angiogenic	Serum	ELISA	Promotes blood vessel formation in the placenta	^234^
2.	Soluble Fms-like Tyrosine Kinase-1 (sFlt-1)	Antiangiogenic	Whole blood	ExoQuick (System Bioscience, Inc., SBI, Mountain View) precipitation method	Inhibits the activity of PlGF and VEGF, leading to endothelial dysfunction	^235^
3.	Vascular Endothelial Growth Factor (VEGF)	Pro-angiogenic	Serum	ELISA	Stimulates the formation of blood vessels, essential for placental development	^234^
4.	Endoglin (sEng)	Co-receptor	Whole blood	ExoQuick (System Bioscience, Inc., SBI, Mountain View) precipitation method	Modulates angiogenesis and is involved in vascular development	^235^
5.	C19 MC micro RNAs (miRNAs)	Genetic material	Plasma & Placental tissue	mirVana microRNA Isolation kit & Trizol method	Regulate gene expression, potentially impacting placental development and function	^236^
6.	Cell-free DNA (CfDNA)	Genetic material	Plasma	ExoQuick exosome precipitation, ExoLution Plus extraction and differential centrifugation	Reflects placental health and can indicate placental dysfunction	^237^
7.	A Disintegrin and Metalloprotease-12 (ADAM12)	Metalloprotease	Placenta	Mass spectrometry	Involved in placental invasion	^238^
8.	Pregnancy-Associated Plasma Protein A (PAPP-A)	Glycoprotein	Serum	ELISA	Altered levels predict PE	^238^
9.	Interleukin – 6 (IL-6)	Cytokine	Blood	ELISA	Elevated in PE-related inflammation	^239^
10.	Tumor Necrosis Factors (TNF-α)	Inflammatory cytokine	Blood	ELISA	Elevated in PE due to inflammation	^240^
11.	Heat Shock Protein −70 (HSP70)	Heat shock protein	Maternal blood	Western blot	Elevated in cellular stress	^241^
12.	Transthyretin (TTR)	Exosome protein	Placenta	Proteomics/ITRAQ mass spectrometry	Alters placental function	^241^
13.	IL-1β, IL-18	Inflammasome proteins	Placental cells	ELISA	Part of inflammatory cascade	
14.	Soluble Leukemia Inhibitory Factor Receptor (sLIFR)	Glycoprotein	Placenta	Proteomics	Linked to implantation processes	^242^
15.	Angiopoietin-2 (ANG-2)	Angiogenic protein	Maternal serum	ELISA	Blood vessel formation	^243^
16.	Hepatocyte growth factor (HGF)	Growth factor	Maternal blood	Protein multiplex	Promotes vascular growth	^243^
17.	SM C28:1, SM C30:1	Metabolites	Serum	Metabolomics	Predictive metabolites	^244^
18.	Exosome Surface Protein CD63 For GDM	Exosome surface protein	Placental exosomes	Flow cytometry	Reflects cell origin/Increased in PE exosomes	
	**FOR GDM**					
19.	miR-29a/b	Genetic material	Venous blood/serum	Trizol Total RNA Isolation	Decreased expression; potential for prognosis evaluation	^245^
20.	miR-21	Genetic material	Placental samples	Trizol method for extraction & TaqMan MicroRNA Reverse Transcription Kit for quantification	Down-regulated; inhibits cell proliferation and infiltration by inducing PPAR-α	^246^
21.	miR-195-5p	Genetic material	Serum samples	TRIzol LS reagent (Life Technologies)	Upregulated; associated with GDM	^247^
22.	miR-875-5p	Genetic material	blood serum	TRIzol reagent (Invitrogen; Thermo Fisher Scientific)	Regulates insulin resistance and inflammation via targeting TXNRD1	^248^
23.	miR-330-3p	Genetic material	Peripheral blood samples/Plasma	MiRNeasy miRNA extraction kit (Qiagen)	Upregulated in plasma of GDM patients	^249^
24.	miR-132	Genetic material	Blood and placental tissue samples	TRIzol reagent (Invitrogen)	Diagnostic biomarker; regulates trophoblast cell viability	^250^
25.	miR-140	Genetic material	Placental tissues and peripheral blood plasma	TRIzol reagent method (Invitrogen) & Quantitative RT-PCR	Dysregulated; related to defective insulin receptor signaling	^251^
26.	miR-574-5p	Genetic material	Plasma samples	miRNeasy Serum/Plasma kit (Qaigen)	Potential metabolic regulator for serum lipids and blood glucose	^252^
27.	miR-181d	Genetic material	Serum samples	TRIzol reagent (Thermo Fisher Scientific, USA)	Promotes pancreatic β cell dysfunction by targeting IRS2	^253^
28.	miR-16, -29a, -134	Genetic material	Blood/serum samples	TRIReagent LS (Sigma-Aldrich) & Nanodrop quantification	Early identification markers for GDM	^254^
29.	Hs-CRP and SHBG	Proteins	Venous blood samples	ELISA quantification	Hs-CRP—upregulated; SHBG—downregulated among patients who developed GDM	^255^
30.	hsa_circRNA_0039480	Genetic material	Peripheral blood samples	Microarray & Western blot	Highly expressed in GDM; may serve as a biomarker for early diagnosis of GDM	^193^
31.	Adiponectin	Glycoprotein	Serum	ELISA	Decreased in GDM	^256^
32.	Sex hormone-binding globulin (SHBG)	Hormone-binding protein	Blood serum	Immunoassay	Lower levels predict GDM	^256^
33.	Insulin	Peptide hormone	Maternal blood	ELISA	Elevated levels in GDM	^257^
34.	sCD163	Soluble glycoprotein	Macrophages	ELISA	Immune response modulation	^257^
35.	Uterine artery pulsatility index (UtA-PI)	Doppler ultrasound measurement	Uterine arteries	Doppler ultrasound	Blood flow assessment	^257^
36.	Body Mass Index (BMI)	Predictor	Baseline measurement	Statistical modeling	High BMI linked to GDM risk	^257^
37.	HSP27, HSP60	Heat shock proteins	Placenta	Western blot	Elevated in GDM-related stress	^258^
38.	IL-6, CRP	Inflammatory markers	Maternal serum	ELISA	Elevated due to GDM	^239^
39.	Visfatin	Adipokine	Maternal blood	ELISA	Metabolic regulation	^259^
40.	FGF21	Adipokine	Maternal blood	ELISA	Regulates metabolism	^259^
41.	Hemoglobin A1c (HbA1c)	Glycoprotein marker	Blood	ELISA	Higher levels indicate GDM	^260^
42.	Oral Glucose Tolerance Test (OGTT)	Glycemic; Glucose tolerance indicator	Blood sample	Plasma glucose measured at intervals after glucose ingestion	Gold standard for diagnosing GDM, assessing glucose metabolism under stress	^261^
43.	Fasting Plasma Glucose (FPG)	Glycemic; Glucose indicator	Blood sample	Enzymatic colorimetric assays, point-of-care glucose meters	Elevated FPG indicates impaired glucose tolerance; used as a key diagnostic criterion for GDM	^261^
44.	Leptin	Hormonal; Cytokine-like protein	Adipose tissue	ELISA, radioimmunoassay	High levels are related to increased insulin resistance and metabolic imbalance in GDM	^262^
45.	Exosomal Glypican-1 (GPC1)	Cell surface glycoprotein; Exosome marker	Plasma exosomes	Western blot, flow cytometry	Linked with insulin sensitivity and β-cell function, with elevated levels in GDM	^263^
46.	Fetuin-A	Glycoprotein; Exosome-associated protein	Blood, plasma exosomes	ELISA, Immunoblotting	Higher levels are associated with insulin resistance and inflammation, contributing to GDM progression	^264^
47.	Adipocyte Fatty Acid-Binding Protein (AFABP)	Lipid-binding protein; Adipokine	Blood, exosomes	ELISA, mass spectrometry	Higher levels correlate with increased lipolysis and metabolic dysregulation in GDM	^265^
48.	Resistin	Inflammatory protein; Exosome-associated	Adipose tissue, plasma exosomes	ELISA, Immunoblotting	Elevated levels in GDM; promotes insulin resistance and inflammation	^266^
49	Endoglin (CD105)	Glycoprotein; Involved in angiogenesis	Placenta, plasma exosomes	ELISA, flow cytometry	Dysregulated in GDM, contributing to endothelial dysfunction and vascular changes in the placenta	^267^
50.	Omentin-1	Adipokine; Exosome-associated protein	Blood plasma, adipose tissue	ELISA	Reduced levels are associated with insulin resistance and inflammation in GDM	^268^
51.	Plasminogen Activator Inhibitor-1 (PAI-1)	Fibrinolytic system protein; Serine protease inhibitor	Plasma	ELISA, immunoassays	Elevated in GDM, linked to hypercoagulability and endothelial dysfunction	^269^
52.	Lipocalin-2 (LCN2)	Iron-binding protein; Secreted adipokine	Plasma, placenta	ELISA, Immunoblotting	Elevated in GDM, linked to inflammation, iron metabolism, and insulin resistance	^270^
53.	Endothelin-1 (ET-1)	Vasoconstrictor peptide; Endothelial-derived	Blood vessels, plasma	ELISA, immunohistochemistry	Increased in GDM, contributes to vascular dysfunction and hypertension	^271^
54.	Asprosin	Glucogenic protein; Circulating hormone	Plasma	ELISA, mass spectrometry	Elevated in GDM, involved in hepatic glucose release and insulin resistance	^272^
55.	Oxidized LDL (ox-LDL)	Lipoprotein; Oxidative stress marker	Blood plasma	ELISA, lipid peroxidation assays	Higher levels are linked to oxidative stress and endothelial dysfunction in GDM	^273^

### 8.1 Ultracentrifugation vs Size-Exclusion Chromatography

Due to the uncomplicated protocol and ease of processing large volumes, Ultracentrifugation (UC) has been considered as the benchmark for exosomes. However, the method is restricted by its coisolation of protein aggregates and extracellular vesicles of similar density. UC, when combined with polymer precipitation, significantly enhances the yield but at the cost of purity, as protein contaminants are often retained as described by Shami-Shah et al.[Bibr ref181] 2023. To tackle this issue, size-exclusion chromatography (SEC) has been proposed as an alternative, as it aids in exosome separation depending on its molecular size while preserving its structural integrity. Mitchell et al. 2022 compared SEC to UC and revealed that while SEC offers remarkable purity, it is limited by its incapacity to process high sample volumes efficiently, making it more suitable for research than clinical-scale applications.^182^ These findings suggest that combining UC and SEC (where UC is used for bulk separation and SEC for refinement) could create a hybrid approach that maximizes both yield and purity.

### 8.2 Immuno-Affinity Capture

Immuno-affinity-based capture (IAC) methods have been engineered to improve specificity by targeting surface markers of exosomes, viz., CD9, CD63, and CD81. As demonstrated by Gorgzadeh et al. 2024, IAC offers superior specificity in extracting tumor-derived exosomes, which is a crucial aspect of biomarker discovery.^183^ The main drawback of this technique is its high dependency on the attainability of specific antibodies and is cost-prohibitive for large-scale applications. Zhou et al. 2024 addressed these challenges by incorporating SEC with IAC, thus extrapolating the SEC’s ability to dislodge protein contaminants before applying antibody-based capture, thereby increasing both purity and specificity.^127^ This suggests that although IAC is a powerful tool for highly specific applications, it benefits from preprocessing steps that remove bulk contaminants.

### 8.3 Microfluidic-Based Isolation

The time-consuming nature and low throughput are the major limitations of traditional methods. To tackle these parameters, an electro-kinetically empowered microfluidic dependent for exosome isolation technique was reported by.^184^ This approach enables rapid, label-free, and high-purity separations, making it specifically suitable for point-of-care (POC) diagnostics. Similarly, Yaman et al. 2025 have come up with EV-Lev tech, which is a microfluidic magnetic levitation device that not only enhances selectivity but also enables high-throughput processing, which was lacking in traditional methods.^185^ Although microfluidic platforms in exosome research are emerging as robust tools, they still face challenges in terms of standardization and scalability. To bridge this gap, Chernyshev et al. 2023 proposed a bead-assisted microfluidic isolation system, integrating IAC with microfluidic processing to enhance both specificity and throughput. The convergence of microfluidics with established isolation techniques signals a shift toward automated, high-efficiency protocols that possess the potential to redefine exosome isolation especially in bed-side and clinical settings.^186^

Furthermore from a diagnostic perspective, Prostatic Acid Phosphatase (PLAP)- ELISA kits are readily accessible for characterizing placental EVs and achieves 92% sensitivity and 88% specificity for preeclampsia detection, significantly outperforming traditional ELISA, which reports 75% sensitivity and lower specificity due to nonexosomal protein background noise.^182^ An immunocapture technique has also been reported for enriching PLAP-positive EVs from maternal circulation and the direct enrichment of PLAP + EVs from blood using gold-loaded nanoporous nanocubes. A hybrid approach of Immunocapture-based microfluidic platforms targeting placental exosome markers (CD63, CD81) exhibits 95% specificity for gestational diabetes detection, drastically reducing false positives compared to conventional immunoassays (80%).^187^ A recent study by Mitchell and co-researchers developed the ultrasensitive immuno-purification assay, termed EV-CATCHER, with a monoclonal antibody targeting the membrane PLAP protein.^188^

Preeclampsia affects almost 10 million females worldwide,^189^ indicating the extent of pregnancy risks. It is one of the intricate pregnancy disorders, typically attributed as a disease associated with chronic hypertension, with a distinction in its onset and nature. It comprises a complex multisystem syndrome with a notable difference in its pathological and pathophysiological aspects in contrast to chronic hypertension.^190^ The typical symptoms include a high blood pressure reading of 140/90 mm of Hg twice in a 4-h gap, proteinuria, thrombocytopenia, headache, temporary loss/alteration of vision, pulmonary edema, stroke, nausea/vomiting,^11^ etc. Primarily, pregnant women who are suspected of preeclampsia are subjected to hematourological tests to detect symptoms as discussed above, and the cases are clinically confirmed by Doppler assessment. However, at times, some women might have the possibility of ambiguous diagnosis due to nonspecific abnormalities.

At this juncture, biomarkers are crucial in enhancing diagnostic accuracy and getting confirmation. The burden of GDM is also no less than close to 1 million hyperglycemia-associated cases in pregnant women.^30^ In some lower-middle-income countries in Africa and Asia, epidemiological investigations reveal that the prevalence of GDM ranges from 1–30% and has become a pervasive pregnancy-associated disorder alongside Preeclampsia globally.^191–193^ GDM is fundamentally a metabolism-associated disease that is clinically diagnosed at around the fifth–sixth month of pregnancy, and the hyperglycemic condition further elevates the case complexity, leading to obvious symptoms like heart-related abnormalities, obesity and even becoming a cocause for Preeclampsia, thus affecting the mother and developing fetus by the time diagnosis is confirmed.^194^ Early prognosis of the disease in such cases is made possible by clinically investigating the exosome biomarker properties. The latest literature has further validated the impact of exosomal miRNAs (Exo-miRNA) as prime indicators for pregnancy complications (Figure 7). Fóthi et al., 2024 identified specific miRNA signatures in maternal blood during the first trimester, which could prognosticate gestational diabetes and preeclampsia.^195^ In a study, it was shown that placental exosomal miR-520a-5p levels were drastically elevated in severe preeclampsia and intrauterine growth restriction (IUGR), and proteomic profiling of maternal exosomes identified 855 differentially expressed proteins, distinguishing normal from high-risk pregnancies.^196^ This data accredits the feasibility of exosomal biomarkers for early first-trimester detection of pregnancy complications.^197^ Another instance in Antiphospholipid syndrome (APS), an autoimmune disorder associated with pregnancies, shows distinct exosomal miR-499 expression, impacting NF-κB pathway activity.^198^ All of these new revelations extensively suggest that exosome profiling could serve as a benchmark for preemptive diagnosis of high-risk pregnancies. Although the importance of exosome research as potential biomarkers in clinical diagnosis is rapidly evolving, they are still under extensive lookout. Further research is highly desired to decipher and understand the complex cargo at different stages of pregnancy and its underlying mechanisms to get a clear picture of pregnancy-associated disorders.

**Figure 7:**
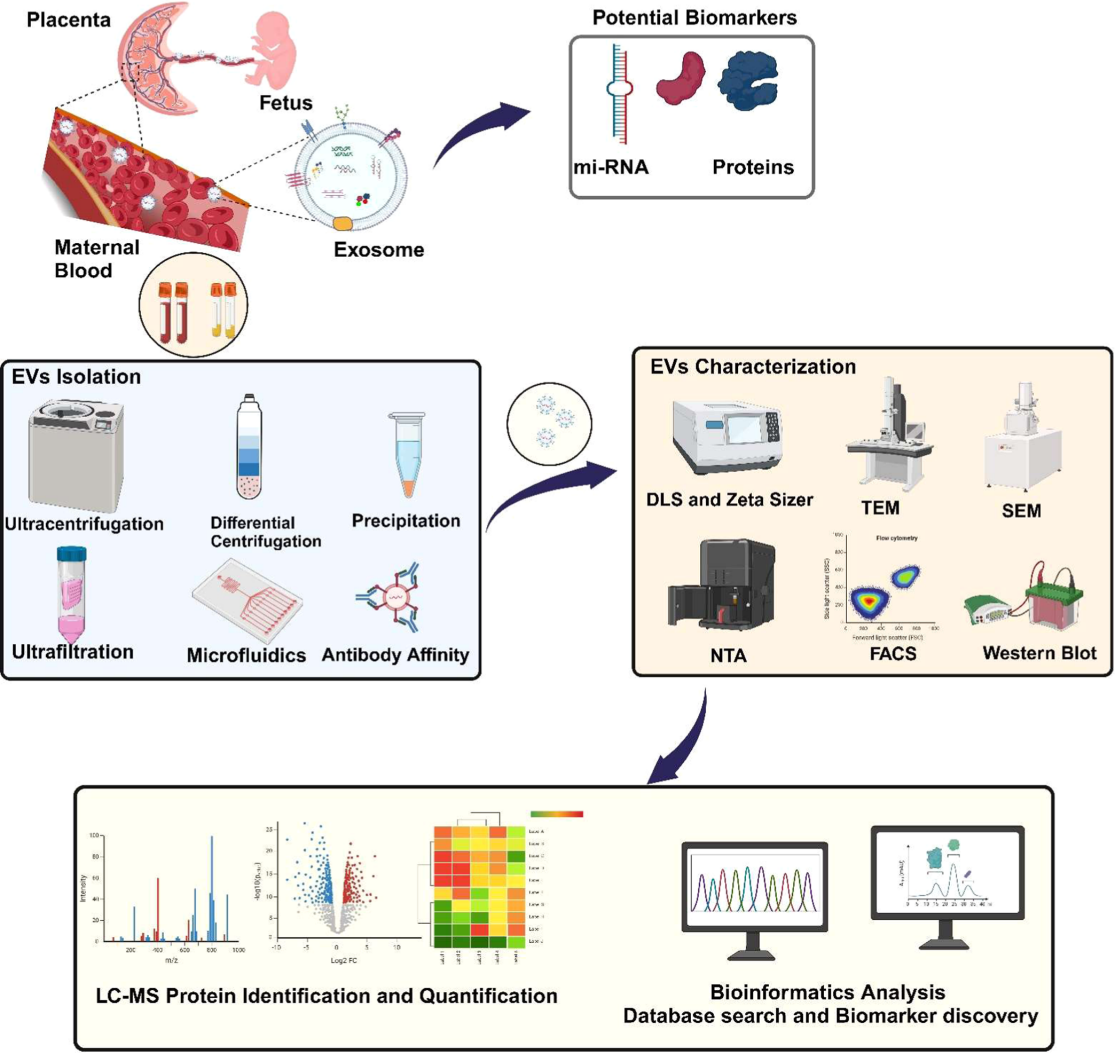
Schematic representation of workflow placental exosome isolation with different available conventional as well as modern methods and biomarker detection (created with Biorender.com).

Clinical validation of exosomal biomarkers in well-designed large cohort studies has been increasingly relevant for the development of noninvasive, early diagnostic tools for these diseases. New studies have emphasized the reliability of exosomal microRNAs (miRNAs), proteins, and lipidomic signatures in prognosing such conditions, thus strengthening their translational potential. To authenticate the clinical relevance of exosome, a large-scale retrospective case-control study was done by Hromadnikova et al. 2019 consisting of 4356 singleton pregnancies in the Caucasian population.^139^ Maternal plasma samples were collected between 10–13 weeks of gestation, and exosomes were isolated to conduct an extensive analysis of the C19MC microRNA cluster, which showed considerable differential expression patterns in women who later developed gestational hypertension, preeclampsia, or fetal growth restriction. The study reported that miR-517-5p, miR-518b, and miR-520h were downregulated, with predictive performance showing sensitivities ranging from 82 to 88% and specificities between 80 and 85%, positioning these miRNAs as reliable first-trimester biomarkers. Another retrospective nested case-control study in a cohort of 3,600 women undergoing first-trimester integrated screening by employing fetal nuchal translucency ultrasound, serum PAPP-A, and β-HCG measurements between 11–13+6 weeks of gestation as conducted by Xu et al. 2024.^197^ Placental exosomal miR-520a-5p levels were measured using qRT-PCR, revealing a statistically appreciable rise in women who, in due course, developed severe preeclampsia and IUGR. The predictive efficacy, assessed through ROC analysis, illustrated an AUC of 0.806 (*p* < 0.001), emphasizing its potential in early clinical screening models.^197^ In a distinct spectrum of complications was investigated by Sun et al. 2020 for exosomal miRNAs as clinical hits for ectopic pregnancy through a prospective validation study of 36 women presenting with early pregnancy symptoms. Differential expression analysis recognized exosomal miR-378d, miR-100-5p, and miR-215-5p to be significantly upregulated in ectopic pregnancy cases. A panel for a collective of hCG, progesterone, and the identified miRNAs showed a remarkable specificity of 80% at a sensitivity of 91%, thus demonstrating promising diagnostic application in acute care settings.^199^ All these multicohort validations emphasize that exosome-based biomarkers hold great promise for early, noninvasive detection of pregnancy complications as predictive clinical models.

## 9 Challenges and Future Perspectives

With significant research being conducted on exosome applications, it is critical to understand the progress made and the ongoing challenges. Although EV analysis has advanced significantly in recent decades, the exact mechanisms of biogenesis remain unknown. Exosomes in maternal circulation come from a variety of tissues, including the placenta, immune cells, and endothelium. Distinguishing between these sources is difficult but necessary as placenta-derived exosomes are more predictive of pregnancy status. Current methodologies lack the precision to accurately trace exosome origins, complicating the interpretation of biomarker data.^196^ Researchers face numerous challenges in understanding the exact mechanisms of exosomes in pregnancy complications. The main challenge is using an experimental approach to understand the processes that occur within the human body. Another issue is related to the separation of placental exosomes from maternal circulation. Purified exosome isolation requires a well-established, standard workflow.^38^ Exosome production and extraction on a large scale with a high purity are still essential. It is crucial to develop an affordable technology for clinical use. Further research is also required on delivery techniques (e.g., oral, intravenous, and intraperitoneal), and repeated exosome administration’s long-term safety (toxicity, immunogenicity) has to be examined. Creating reliable diagnostic tools with placental exosomes requires the standardization of isolation and analysis techniques. Exosomes have attracted attention as possible guides for identifying and forecasting infertility issues as theory is essential for controlling the process of embryo implantation. Placental exosomes may act as disease-predictive indicators as a result of this early discovery, allowing medical professionals to create prompt treatment plans. The ability of placental exosomes to be isolated from mother blood offers a non-invasive way to track placental health, which is advantageous when employing them.^81^ However, with a number of encouraging studies, this discipline is still in its infancy; therefore, further research is still required to establish the reliability and specificity of placental exosomes as predictive markers. The potential of exosomal mRNA, miRNAs, and nucleic acid sequences as possibilities for forecasting the start of illness has been emphasized by ongoing investigations. Nevertheless, further research and validation are required to convert these candidates into trustworthy biomarkers for determining the likelihood of developing pregnancy-related problems.

Furthermore, the prognosis and progression of PE are correlated with different levels of exomiR expression. The investigation of EVs in GDM has the potential to improve our comprehension of the disease’s causes and provide diagnostic instruments.^200^ Even with this advancement, there are still major obstacles in the way of EVs being clinical diagnostic tools rather than just research discoveries. Numerous EV sources, including urine, plasma, cultured explant medium, and different methods for EV separation and characterization, have been employed in the studies. Additionally, research has investigated the relationship between EV alterations and clinical factors, frequently focusing on small cohorts and particular groups. EVs may have lasting impacts that contribute to long-term issues. Without large-scale replication trials to establish clinical effectiveness and extrapolate the potential, most research has now included a small cohortset. To help manage this problematic illness, international research teams devoted to exosomes should prioritize this field of study. Exosomes are safer as biological products because of their ability to avoid being phagocytosed or destroyed by macrophages because of their tiny size and biological activity.^201^ However, because of their uncertain nature and activity, it is still difficult to anticipate the long-term safety and therapeutic impact of these biomarkers.

Despite studies revealing that free β-human chorionic gonadotropin (f-hCG) is unsuitable for PE prediction due to consistent findings across 10 studies showing no significant difference in f-hCG levels between PE cases and controls, other markers like ADAM12 and activin A remain inconclusive with contradictory or limited data.^202^ This necessitates further studies to ascertain their potential role in PE screening. Conversely, significantly reduced concentrations of placental protein 13 (PP13), placental growth factor (PlGF), and pregnancy-associated plasma protein-A (PAPP-A) in the first trimester, along with increased levels of inhibin A, have been strongly linked to the onset of PE. Nonetheless, none of these serum markers appear promising due to only modest detection rates (DRs) at a false positive rate (FPR) of 10%. Thus, single marker screening is deemed unsuitable for clinical practice, and combining the best-performing serum markers with maternal constitutional characteristics and/or uterine artery (Ut-A) Doppler assessments yields higher DRs, making them more promising.^203^ This approach is especially relevant for early-onset PE, which is linked to numerous maternal and fetal complications. Large prospective studies are needed to confirm these associations and validate the potential utility of marker combinations in various populations. Exploration into other markers, such as nucleic acids, proteins, peptides, and cellular metabolites, is ongoing. Unlike Doppler ultrasound, which relies on operator skill and standardized measurement techniques, exosomal biomarkers can offer more consistent and reproducible results and real-time insights into cellular processes and pathophysiological changes occurring in the placenta. By integrating exosomal biomarkers with traditional screening methods, it is possible to overcome limitations associated with Doppler ultrasound, potentially enhancing early detection of pregnancy-associated risks and improving maternal and fetal outcomes.

## 10 Conclusions

Maternal mortality and pregnancy-related complications present significant global health challenges, severely impacting the health of both mother and baby. These complications are exacerbated by maternal illnesses, with key causes and modifiable risk factors including infections, hypertension, congenital malformations, fetal growth restriction, and prematurity. Therefore, enhancing maternal care is crucial to minimizing these risks. Improving fetal outcomes requires the early identification of potential risk factors and the implementation of effective perinatal management strategies, particularly focusing on the early detection of fetal growth restriction. Currently, diagnoses for these conditions rely on conventional screening or diagnostic methods, and there is a shortage of biomarkers with an early predictive value. The development of noninvasive diagnostic techniques has highlighted the potential of exosomes in the peripheral blood of pregnant women as biomarkers for pregnancy-related diseases. Exosomes, which are instrumental in maternal–fetal interaction during gestation, reflect the microenvironment and metabolic state of their cell of origin, providing insights into these cells’ function and metabolic status. Studies have indicated that the placenta secretes exosomes into the maternal bloodstream, which plays a crucial role in modulating immune responses during pregnancy and maintaining maternal vascular health. Gaining more insight into how exosomes influence important pregnancy processes could shed light on the mechanisms of communication between the mother and fetus under both healthy and abnormal pregnancy conditions. As the field of EVs continues to grow, better characterization of placental exosomes released under various physiological and pathological conditions will be crucial. Understanding the mechanisms of placental exosome transfer between cells could lead to the development of new diagnostic tools and treatments. With rapid advancements in medical science and technology, collecting comprehensive data throughout pregnancy and various types of samples will aid in the development of predictive models for pregnancy complications and abnormal fetal development. Comprehensive analysis using advanced techniques is vital for answering unresolved questions, testing hypotheses, and exploring potential treatment options for pregnancy disorders. Enhanced analytical methods are needed to address open questions, pursue intriguing hypotheses, and explore potential therapeutic avenues for pregnancy pathologies.
